# Construction and validation of a transmembrane 4 superfamily-related genes prognostic model for esophageal squamous cell carcinoma

**DOI:** 10.3389/fonc.2025.1580199

**Published:** 2025-11-19

**Authors:** Xin Hao, Hao Ding, Hongyu Zhu, Xiaoying Gong, Yuxin Chang, Chunhua Liu, Sicong Hou

**Affiliations:** 1 Department of Gastroenterology, Baoying People’s Hospital, Yangzhou, China; 2 Key Laboratory of Jiangsu Province University for Nucleic Acid & Cell Fate Manipulation, Faculty of Medicine, Yangzhou University, Yangzhou, China; 3 Department of Thoracic Surgery, Baoying People’s Hospital, Yangzhou, China; 4 Department of Radiology, Baoying People’s Hospital, Yangzhou, China; 5 Key Laboratory of the Jiangsu Higher Education Institutions for Integrated Traditional Chinese and Western Medicine in Senile Diseases Control, Faculty of Medicine, Yangzhou University, Yangzhou, China; 6 Department of Gastroenterology, Affiliated Hospital of Yangzhou University, Yangzhou University, Yangzhou, China

**Keywords:** esophageal squamous cell carcinoma, transmembrane 4 superfamily, prognostic model, bioinformatics analysis, immune infiltration

## Abstract

**Background:**

Esophageal squamous cell carcinoma (ESCC), a virulent form of cancer, markedly diminishes prospects for patient survival. The transmembrane 4 superfamily (*TM4SF*)-related genes (TRGs) are instrumental in the advancement and spread of cancer. The intent of the current research was to create a prognostic model for ESCC, grounded in the expression patterns of TRGs;

**Methods:**

The datasets pertaining to ESCC from The Cancer Genome Atlas (TCGA)-ESCC and the GSE53622 cohort were meticulously examined. Differential and regression analyses discerned the pivotal signature genes. Subsequent stratification of patients into distinct risk groups was achieved by employing optimal risk score thresholds. This prognostic precision of model was assessed with Kaplan-Meier (K-M) curves and receiver operating characteristic (ROC) analyses. A nomogram integrating risk score with clinicopathological characteristics was meticulously constructed and subsequently validated. Additional analyses included functional enrichment, immune infiltration, immunotherapy responses, drug sensitivity, and molecular network analysis. The expression levels of the characteristic genes were meticulously examined in both TCGA-ESCC datasets and patient-derived tissues;

**Results:**

24 candidate genes were identified. Among these, *TSPAN15*, *TSPAN9*, and *TSPAN16* were selected as signature genes. The model showed high prediction accuracy via K-M and ROC curves. Prognostic evaluations have indicated that the risk score and the stage of the tumor are pivotal prognostic indicators. The high-risk cohort exhibited elevated dysfunction scores, suggesting a potentially more favorable response to immunotherapy. Significant drug sensitivity differences were observed. *GATA2* regulated all three signature genes, with *TSPAN15* and *TSPAN16* downregulated and *TSPAN9* upregulated. These findings were consistent with RT-qPCR and immunohistochemical results;

**Conclusions:**

*TSPAN15*, *TSPAN9*, and *TSPAN16* are *TM4SF*-related signature genes with prognostic value for ESCC, providing a theoretical foundation for its diagnosis and treatment.

## 1 Introduction

Esophageal cancer (EC) ranks as the 7th most common form of malignancy across global, and it stands as the sixth primary source of cancer-related deaths, exerting a considerable toll on human health. The latest global cancer statistics for 2020 reported 604,000 new cases of EC and 544,000 deaths. It is worth mentioning that the prevalence of this condition is most prominent in Eastern Asia, with China showing the highest incidence rate (1). Histopathologically, Esophageal cancer is primarily categorized into two distinct types: esophageal squamous cell carcinoma (ESCC) and esophageal adenocarcinoma (EAC). In China, ESCC prevails as the predominant subtype, comprising 90% of all EC cases (2), with cigarette smoking and alcohol intake identified as key risk factors (3, 4). Despite the advancements in multimodal treatments for ESCC, the five-year survival rate remains at a disheartening 20% (4, 5). An accurate assessment of ESCC prognosis is critical for clinicians to devise suitable treatment plans and enhance the survival prospects of patients. The prognosis for ESCC is primarily evaluated using TNM staging (6, 7). However, the predictive accuracy of TNM staging alone is limited (8, 9). Therefore, reliable biomarkers and effective models are urgently needed to predict ESCC prognosis and guide therapeutic strategies.

The transmembrane 4 superfamily (*TM4SF*) comprises a set of evolutionarily preserved proteins, distinguished by their four integral transmembrane segments (TM1-TM4), twin extracellular loops (EC1 and EC2), and a solitary intracellular loop, all of which are embedded within the membranes of eukaryotic cells (10, 11). The EC2 domain is mainly responsible for interacting with various tetraspanin molecules and other non-tetraspanin proteins. EC2 disulfide crosslinks help stabilize the tetraspanin structure by stabilizing the TM domains. A total of 33 classical tetraspanins (*TSPAN1*-*TSPAN33*) (12) and 8 newly identified tetraspanins (*TM4SF1*, *TM4SF4*, *TM4SF5*, *TM4SF10*, *TM4SF11*, *TM4SF18*, *TM4SF19*, *TM4SF20*) from the *TM4SF* family have been documented (13, 14). These proteins are instrumental in the initiation and progression of various human malignancies, among which includes ESCC. Studies indicate that *TSPAN27* may act as a suppressor of invasion and metastasis in ESCC by modulating TGF-β1 signaling pathway (15–17). Our previous study identified a novel mechanism by which *TM4SF1* facilitates ESCC metastasis through its interaction with integrin α6 (18). The research indicates that *TM4SF* holds promise as the prognostic pointer and therapeutic target for ESCC. Nonetheless, there currently exist only a limited number of reliable prognostic models that utilize *TM4SF*-related genes (TRGs) to forecast the outcome for patients with ESCC. Thus, it is imperative to cultivate novel and dependable *TM4SF*-associated predictive biomarkers for assessing the correlation between TRGs and patient outcomes, and to guide this understanding of their development and associated immune effects in ESCC. Recent studies have shown that the tumor microenvironment (TME), particularly its immune components, plays a decisive role in shaping therapeutic outcomes. For example, AI-driven mutation signatures and plasma cell-based immune classifiers have been developed to predict immunotherapy responses across cancer types, offering key insights into tumor-immune interactions (19, 20). Moreover, Ye et al. (21) developed an integrated Machine Learning and Genetic Algorithm‐driven Multiomics analysis (iMLGAM), which highlights the prognostic value of immune-related gene signatures and tumor immune infiltration. These findings, although not specific to ESCC, highlight the prognostic relevance of immune landscape features and support the rationale for exploring the immunological implications of TRGs in ESCC.

**Figure 1:**
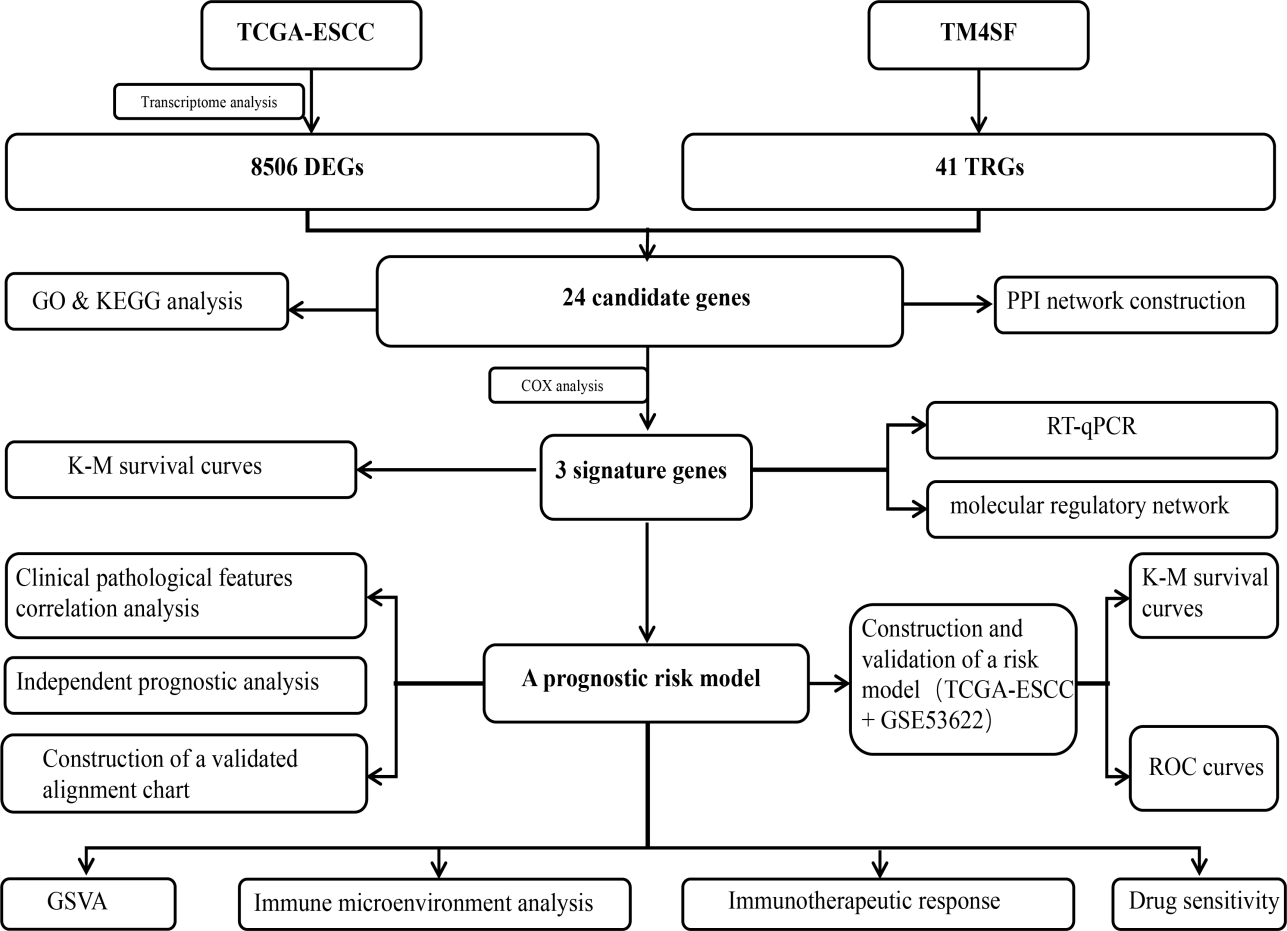
The workflow of the present study.

To address this issue, we analyzed the TRGs in ESCC. We crafted a predictive ESCC risk model utilizing datasets from Gene Expression Omnibus (GEO) in conjunction with University of California, Santa Cruz (UCSC) Xena databases. Our study aimed to provide new insights into the pathogenesis of ESCC. The workflow of this study is shown in **Figure 1**.

## 2 Materials and methods

### 2.1 Data source

ESCC-related datasets, The Cancer Genome Atlas (TCGA)-ESCC and GSE53622, were obtained from UCSC Xena (https://xenabrowser.net/datapages/) database and GEO database (https://www.ncbi.nlm.nih.gov/geo/), respectively. The TCGA-ESCC dataset contained count, FPKM, phenotype, and survival data from 162 tumor and 11 normal samples. A total of 251 TCGA-ESCC samples were initially retrieved. After excluding 89 cases with non-squamous cell carcinoma histology, 162 ESCC samples remained. Among these, 81 cases lacking survival information were further excluded, resulting in a final analytic cohort of 81 ESCC samples with complete survival data. The detailed selection process is presented in **Supplementary Figure S1**. GSE53622 (platform: GPL18109) included 60 ESCC patients and was used to validate the risk model. Additionally, 41 TRGs were derived from a previous study (22).

### 2.2 Differential, enrichment analyses and construction of protein-protein interaction network

Differentially expressed genes (DEGs) were discerned among ESCC and adjacent normal tissues within TCGA-ESCC dataset by employing DESeq2 (version 1.34.0) (23), with criteria of *P* value < 0.05 and |log_2_FC| >0.5. Next, DEGs were intersected with TRGs to pinpoint potential candidate genes. Pathway and functional enrichment analyses were conducted using the clusterProfiler package (version 4.2.2) (24) to probe into these underlying biological processes and pathways linked to these candidate genes, with a particular emphasis on unveiling Gene Ontology (GO) and Kyoto Encyclopedia of Genes and Genomes (KEGG) databases, applying a stringent significance threshold of *P* < 0.05. The PPI network of candidate genes was constructed via STRING (http://www.string-db.org/) database for evaluating protein-level interactions, with a confidence score of 0.3. Building and validation of risk model.

### 2.3 Building and validation of risk model

Univariate Cox regression analysis was conducted for specify potential prognostic genes linked to survival, utilizing the Coxph function for thorough screening (*P* < 0.2) (25). The proportional hazards (PH) hypothesis test (*P* > 0.05) was then performed, and genes that passed the test were integrated into a comprehensive multivariate Cox regression analysis. We used the Step function within R software for stepwise regression, employing both forward and backward selection strategies. At each step, the model identifies and includes or excludes variables that most significantly improve the model drawing on the Akaike Information Criterion (AIC) for analysis. The iterative procedure persists until the most optimal and stable model is pinpointed, characterized by the lowest AIC value and encompassing the distinctive genes. Subsequent to this, risk scores were computed by employing penalty coefficients in conjunction with the gene expression data for predictive features. A risk model was developed, and the risk score was calculated using the formula: $risk\;score=\sum_{i=1}^{n}(coefi*Xi)$, where Xi represents the relative expression of prognostic signature gene i, and coefi displays Cox coefficient of signature gene i. Based on the risk score calculation formula, the risk values of samples with survival information in TCGA-ESCC were calculated using the R package “survminer” (version 0.4.9) (26). The optimal cutoff value for the risk score was then calculated by the surv cutpoint function, which evaluates the ability of each potential cutoff point to distinguish survival data by calculating different values and identifies the one maximizing the survival time difference as the optimal cutoff. Finally, the ESCC samples with survival information were divided into high and low risk groups according to the optimal cutoff value. Kaplan-Meier (K-M) plots, in conjunction with a log-rank analysis, were employed for evaluating the statistical significance of disparities in survival rates among the distinct expression cohorts of the identified genes. Furthermore, the participants were stratified into different risk categories in accordance with the established threshold values of their risk scores. The reliability and efficacy of the predictive model were assessed through the employment of K-M survival analysis and construction of receiver operating characteristic (ROC) curves over a span of 1 to 3 years, utilizing the survivalROC package (version 1.0.3) for graphical representation. Moreover, identical analytical methods were employed on the validation dataset GSE53622 to substantiate the model’s predictive capabilities. The risk assessment for the validation dataset was initially determined utilizing this risk score equation, wherein these samples were subsequently classified into high-risk and low-risk cohorts in accordance with the most favorable threshold value. Drawing upon the delineation of high- and low-risk cohorts, the reliability and efficacy of this model were stringently verified within the validation cohort through the employment of K-M survival analyses and ROC curve assessments. To further evaluate the predictive accuracy and potential overfitting of the multivariate Cox regression model, we performed an internal validation using the bootstrap method with 1000 resamples. The mean C-index and 95% confidence intervals from these bootstrap samples were calculated to assess the model’s performance on independent data and to estimate the optimism.

### 2.4 Clinical characterization and independent prognostic analysis

To assess the correlation between signature genes and clinical characteristics, we examined the distribution of these genes across various clinical features, including age, tumor stage, and pathological stage. In the training cohort (TCGA-ESCC) and validation cohort (GSE53622), K-M survival curve analyses were undertaken to gauge these survival disparities among various clinical characteristics across distinct risk categories. Moreover, ROC curve analyses were executed to ascertain this model’s predictive accuracy. Subsequently, these risk scores were integrated with the clinical attributes to conduct univariate Cox proportional hazards (PH) analysis, aiming to pinpoint the factors that correlate with patient survival (*P* < 0.05). These factors were then sequentially subjected to PH assumption testing (*P* > 0.05) and multivariate Cox PH analysis (*P* < 0.05). Factors with a P-value of < 0.05 were considered independent prognostic factors. A nomogram was subsequently evolved, integrating these independent prognostic variables, to gauge a likelihood of patient survival over a 1 to 3 year period, utilizing rms package (version 6.5-0) (26). The calibration plot, ROC curves, and decision curve analysis (DCA) were employed to ascertain the nomogram’s validation and precision. To further quantify the prediction error of the nomogram, additional Brier scores at each time point are calculated.

### 2.5 Expression analysis of known ESCC prognostic genes across clinical subgroups

To validate the biological relevance of the risk model, we analyzed the expression patterns of seven established prognostic genes in ESCC—tumor protein p53 (*TP53*), tRNA methyltransferase 5 (*TRMT5*), epidermal growth factor receptor (*EGFR*), kinesin family member 23 (*KIF23*), fat atypical cadherin 1 (*FAT1*), lysine methyltransferase 2D (*KMT2D*), and catenin beta 1 (*CTNNB1*)—across different risk groups (27–30). Wilcoxon rank-sum tests were performed to compare their expression levels between high- and low-risk groups in both the TCGA-ESCC training set and the GSE53622 validation dataset, with a statistical significance threshold of *P* < 0.05. Furthermore, to investigate the association between these genes and key clinical parameters, a heatmap was generated to visualize their standardized expression profiles across all patient samples.

### 2.6 Pathway enrichment analysis

To explore these intrinsic mechanisms that govern these disparities in survival rates, gene set variation analysis (GSVA) was conducted on TCGA-ESCC dataset, aiming to discern the differential pathway activation between the distinct risk cohorts, using the c2.cp.kegg.v2022.1.Hs.symbols.gmt gene set (*P* < 0.05).

### 2.7 Immunological microenvironmental analysis

To assess the impact of characteristic genes on the immune microenvironment of ESCC, this study employed the single-sample gene set enrichment analysis (ssGSEA) method within gene set variation analysis (GSVA) (v 1.42.0) (31) to conduct immune infiltration analysis. Among these, the gene sets used to quantify the infiltration levels of 28 types of immune cells in ESCC tissue samples were derived from a previously published study (32). The disparities in immune-infiltrating cell distributions across various risk groups were determined through the employment of Wilcoxon rank-sum test (*P* < 0.05). These correlations among risk scores, signature genes, and differentially infiltrated immune cells were analyzed. Furthermore, 21 major histocompatibility complex (MHC)-related genes (33) and 64 chemokine-related genes, including their receptors (34), were obtained from previous studies. Differences in these genes between risk groups were calculated via the Wilcoxon test (*P* < 0.05). In addition, immune cycle-related data were extracted from the TIP website (http://biocc.hrbmu.edu.cn/TIP/index.jsp), and ssGSEA scores for each function were calculated using GSVA to explore differences in the scores for active processes of the immune cycle in cancer across varying risk stratifications. Ultimately, we assessed differences in the Tumor Immune Dysfunction and Exclusion (TIDE) score between risk groups (*P* < 0.05), and evaluated the response rates to immunotherapy within various risk groups. The TIDE was a computational tool commonly used to infer immunotherapy efficacy from genomic data, rather than a direct clinical predictive tool (35). To further verify the characteristics of the ESCC immune microenvironment, this study additionally employed the CIBERSORT algorithm for immune infiltration analysis.

### 2.8 Chemotherapeutic drug sensitivity analysis and molecular network construction

Using pRRophetic (v0.5) (36), the half-maximal inhibitory concentration (IC_50_) values for 138 chemotherapeutic drugs were calculated for each tumor sample, and variations in drug IC_50_ values across risk groups were analyzed. To further investigate the regulatory mechanisms of signature genes in ESCC, we predicted the transcription factors (TFs) and miRNAs corresponding to them using the JASPAR database. A TF-mRNA-miRNA network was constructed employing Cytoscape (v3.8.2) (37).

### 2.9 Expression validation of signature genes

The differential expression patterns of characteristic genes for TCGA-ESCC were extracted to compare their expression between ESCC and control samples. Additionally, we collected 5 pairs of ESCC and control samples for reverse transcription-quantitative polymerase chain reaction (RT-qPCR) and immunohistochemistry (IHC) validation from Baoying People’s Hospital. All samples were accumulated from untreated sufferers, and adjacent normal tissues were obtained from areas at least 2 cm away from tumor margins to avoid contamination. Patients with severe comorbidities or prior cancer treatments were excluded to minimize confounding factors. Informed consent was obtained from all participants. The study was granted ethical approval through the Ethics Committee of Baoying People’s Hospital.

Following sample collection, these specimens were promptly immersed in liquid nitrogen for rapid freezing, before being securely transferred to a -80 °C refrigerator to ensure the integrity of RNA and mitigate degradation. Total RNA from 10 samples was extracted using TRIzol reagent (Invitrogen, China) according to the manufacturer’s protocol. RNA concentrations were subsequently quantified with the NanoPhotometer N50. Following this, cDNA was produced over reverse transcription utilizing the SureScript First Strand cDNA Synthesis Kit (Servicebio, China). Eventually, RT-qPCR was supervised utilizing a CFX Connect Thermal Cycler (Bio-Rad, USA). Amplification conditions are provided in **Supplementary Table S1**. The relative quantification of mRNA transcripts was determined utilizing 2^-ΔΔCt^ way. The ΔΔCt values of the RT-qPCR experiment are shown in the **Supplementary Table S2**. All primer sequences are readily accessible within **Supplementary Table S3**.

After sample collection, the samples were fixed by immersion in 4% paraformaldehyde for 24 to 48 hours. The specimens were meticulously embedded within paraffin wax before being carefully sectioned. Ten paraffin-embedded ESCC tissue blocks were sectioned at 3 μm and baked at 64°C for 1 hour. The sections were dewaxed twice in xylene and rehydrated through a graded ethanol series. After blocking with 5% bovine serum albumin (BSA) for 30 minutes, the sections were incubated with primary antibodies against TSPAN15, TSPAN9, and TSPAN16 at 4°C overnight. On the following day, the sections were rewarmed at 37°C and sequentially treated with a reaction enhancer and an enhanced enzyme-labeled secondary antibody, each for 20 minutes. Finally, the sections were developed with 3,3’- diaminobenzidine (DAB), counterstained with hematoxylin, dehydrated through a graded ethanol series, cleared in xylene, and mounted with neutral resin. Ultimately, these samples were spotted, mounted on slides, and scanned. ImageJ and Pro Plus software were used for analysis, while GraphPad Prism was employed for result visualization.

### 2.10 Statistical analysis

All the analytical procedures were executed utilizing the R package (v4.2.2). A P-value of < 0.05 was deemed statistically significant.

## 3 Results

### 3.1 Identification and functional enrichment of candidate genes: a total of 24 candidate genes were identified and subjected to functional enrichment analysis

**Figure 2:**
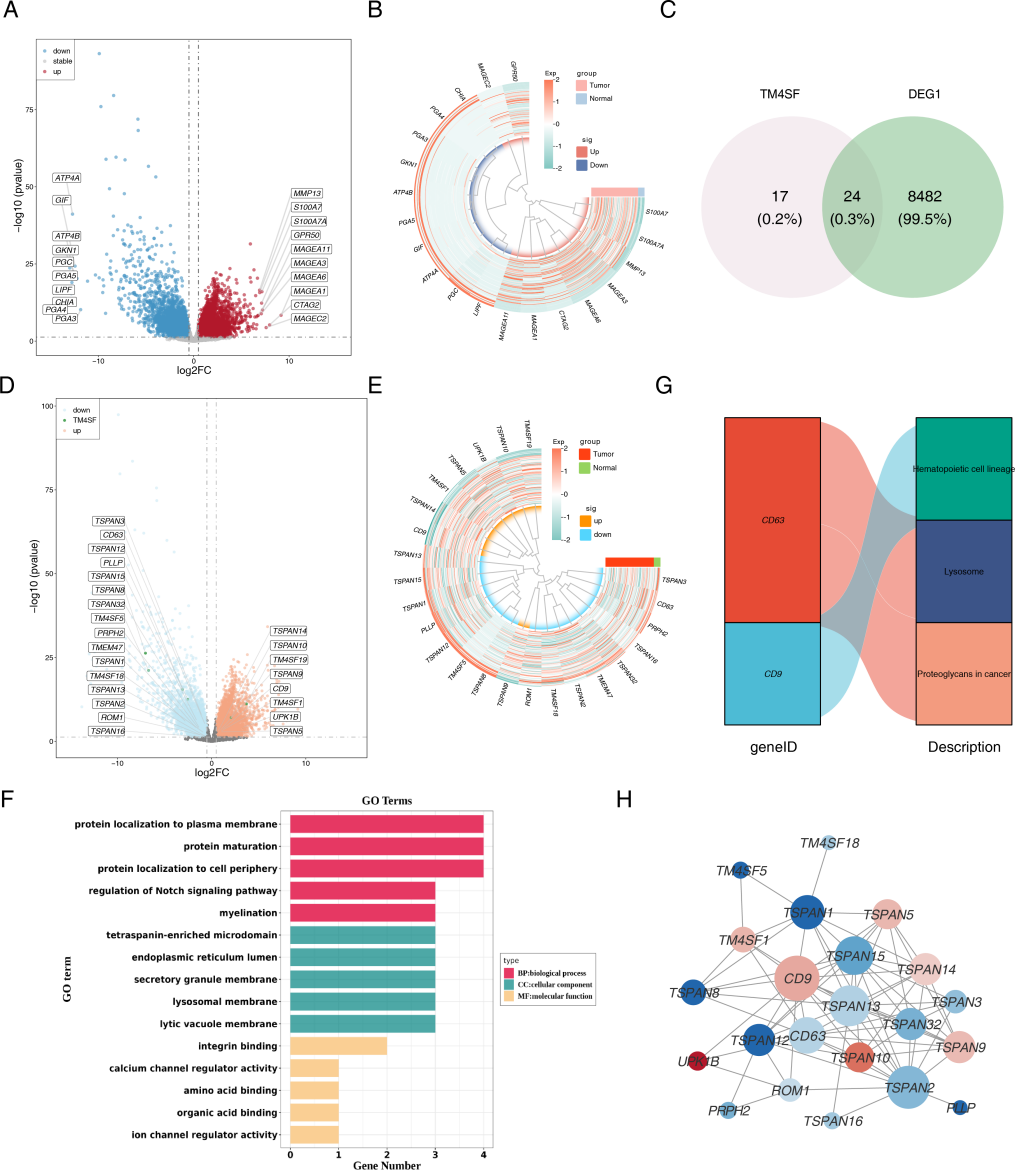
Identification and functional enrichment of differentially expressed TRGs in ESCC patients. **(A)** The volcano plot of DEGs. Red indicated up-regulated genes, blue indicated down-regulated genes, and grey indicated non-significant genes. **(B)** The heatmap of top 10 distinctly up-regulated and down-regulated genes. The colors represented the relative expression of the genes, with red representing relatively high expression and blue representing relatively low expression. **(C)** The Venn diagram of DEGs and *TM4SF*. **(D)** The volcano plot and **(E)** heatmap of candidate genes. **(F)** GO enrichment analysis and **(G)** KEGG pathway enrichment analysis of the 24 candidate TRGs. **(H)** PPI network of candidate TRGs. The nodes represent genes, the size of the dots represents the magnitude of connectivity, and the colors represent up-down relationships.

A total of 8,506 DEGs were discerned between these tumor and normal tissues, with 4,242 genes exhibiting elevated expression and 4,264 genes manifesting reduced expression (**Figures 2A, B**). DEGs were then overlapped with TRGs to identify 24 candidate genes (**Figure 2C**). **Figures 2D, E** display the expression of candidate genes. Subsequent functional and pathway enrichment analyses uncovered that these genes pertained to the modulation of the Notch signaling pathway, integrin binding, ion channel regulator activity, hematopoietic cell lineage, lysosomes, and proteoglycans in cancer (**Figures 2F, G**). Moreover, the PPI network revealed higher connectivity for *CD9*, *TSPAN*2, *TSPAN13*, *TSPAN15*, and *CD63* (**Figure 2H**).

### 3.2 Construction of a risk model with a high accuracy

**Figure 3:**
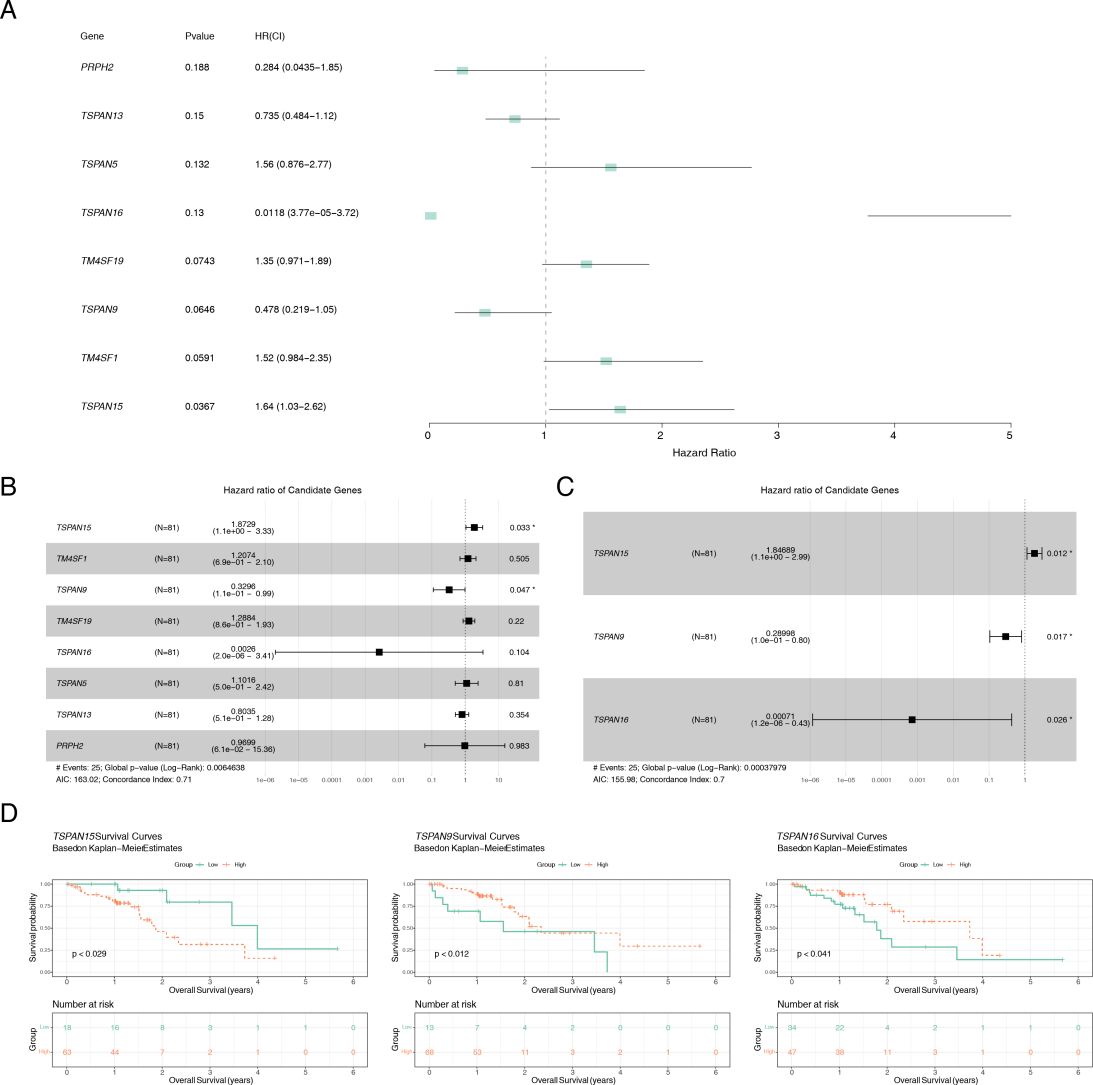
Identification of the signature genes. **(A)** Forest plot of univariate Cox analysis, genes with *P* < 0.2 were defined as associated with prognosis. **(B)** Forest plot of multifactorial Cox analysis based on eight genes associated with prognosis. **(C)** stepwise regression analysis of forest maps to identify signature genes. **(D)** K-M survival curves of patients between high and low expression groups of signature genes. *P < 0.05.

**Figure 4:**
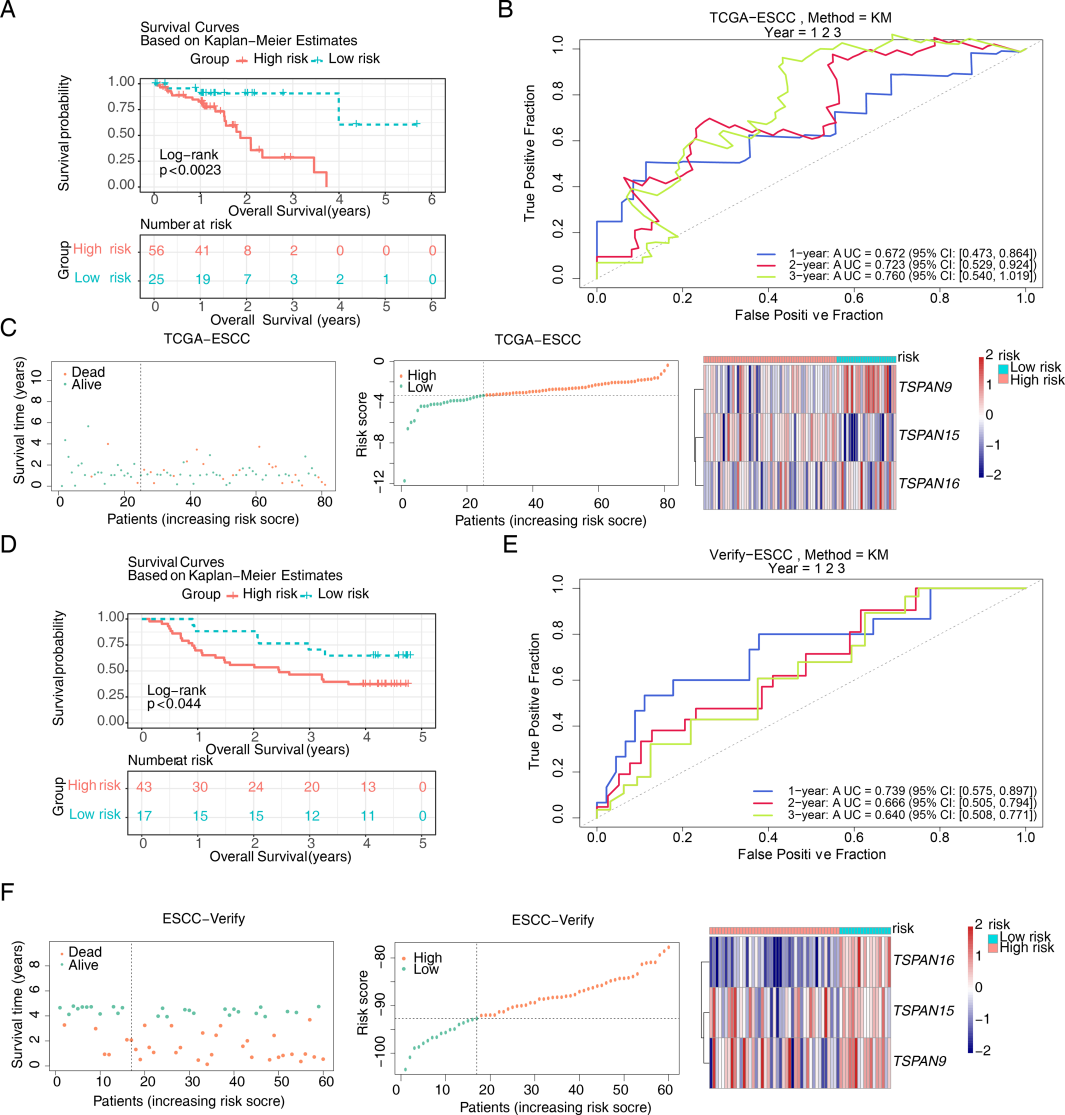
Construction of the risk model based on signature genes. K-M survival curves for patients between high and low risk groups in **(A)** the training set and **(D)** the validation set. ROC curves for patient survival at 1,2,3 years in **(B)** the training set and **(E)** the validation set. The survival status, survival time, and gene expression in **(C)** the training set and **(F)** the validation set. In the distribution plot of patient survival time and survival status, the horizontal coordinates were the samples of patients sorted according to their risk scores, with increasing risk scores from left to right, and the vertical coordinates were the patient survival time and risk scores, respectively. In the heat map of gene expression, red represented higher relative expression and blue lower.

Of the 24 candidate genes, 8 were found to be connected to survival using univariate Cox regression analysis (*P* < 0.2) and successfully passed the PH assumption test (*P* > 0.05) (**Figure 3A**). A multivariate Cox PH model was subsequently developed incorporating an influence of 8 genes (**Figure 3B**), and the optimal model was selected using stepwise regression (*P* < 0.05), which included 3 signature genes: *TSPAN15* (*P* = 0.012, HR = 1.847, confidence interval (CI) = 1.1e + 00 - 2.990), *TSPAN9* (*P* = 0.017, HR = 0.290, CI = 1.0e - 01 - 0.800), and *TSPAN16* (*P* = 0.026, HR = 0.001, CI = 1.2e - 06 - 0.430) (**Figure 3C**). Furthermore, these signature genes significantly differed among high and low-expression groups within K-M survival curves (*P* < 0.029, *P* < 0.012, *P* < 0.041) (**Figure 3D**). In addition, the formula for the risk score is: RiskScore=TSPAN15*(0.614)+TSPAN9*(−1.238)+TSPAN16*(−7.253). Sufferers of TCGA-ESCC were categorized into different risk groups drawing upon the optimal threshold of risk score. The survival analysis revealed that individuals within high-risk cohort exhibited diminished survival probabilities (**Figure 4A**). This model exhibited exceptional prognostic precision for overall survival (OS), achieving area under the curve (AUC) metrics of 0.672 (95% Cl: 0.473-0.864), 0.723 (95% Cl: 0.529-0.924), and 0.760 (95% Cl: 0.540-1.019) for the 1-, 2-, and 3-year survival forecasts, respectively (**Figure 4B**). The Harrell’s C index was 0.691 (95% Cl: 0.542-0.833). Significantly, the survival distribution graphs revealed a progressive rise in mortality rates corresponding to ascending risk scores, with *TSPAN9* being overexpressed in the low-risk cohort and *TSPAN15* exhibiting reduced expression (**Figure 4C**). Likewise, we substantiated the model’s predictive capability within the GSE53622 dataset. The high-risk cohort exhibited consistently poorer survival outcomes, with a statistical significance of *P* < 0.044 (**Figure 4D**), and AUC metrics for 1-year, 2-year, and 3-year predictive intervals for patients were recorded at 0.739 (95% CI: 0.575–0.897), 0.666 (95% CI: 0.505–0.794), and 0.640 (95% CI: 0.508–0.771), respectively (**Figure 4E**), aligning with TCGA-ESCC findings. The Harrell’s C index was 0.625 (95% Cl: 0.530-0.714). The low-risk cohort demonstrated an elevation in the expression of these three pivotal signature genes (**Figure 4F**). The mean C-index derived from the bootstrap method was 0.772 (95% CI: 0.645–0.893), which was higher than the original C-index of 0.707 (95% CI: 0.590–0.750). These results indicated that the model effectively discriminated survival risks among patients and possessed clinical reference value.

### 3.3 Risk score and tumor stage as independent prognostic factors for ESCC and construction of a validated alignment chart

**Figure 5:**
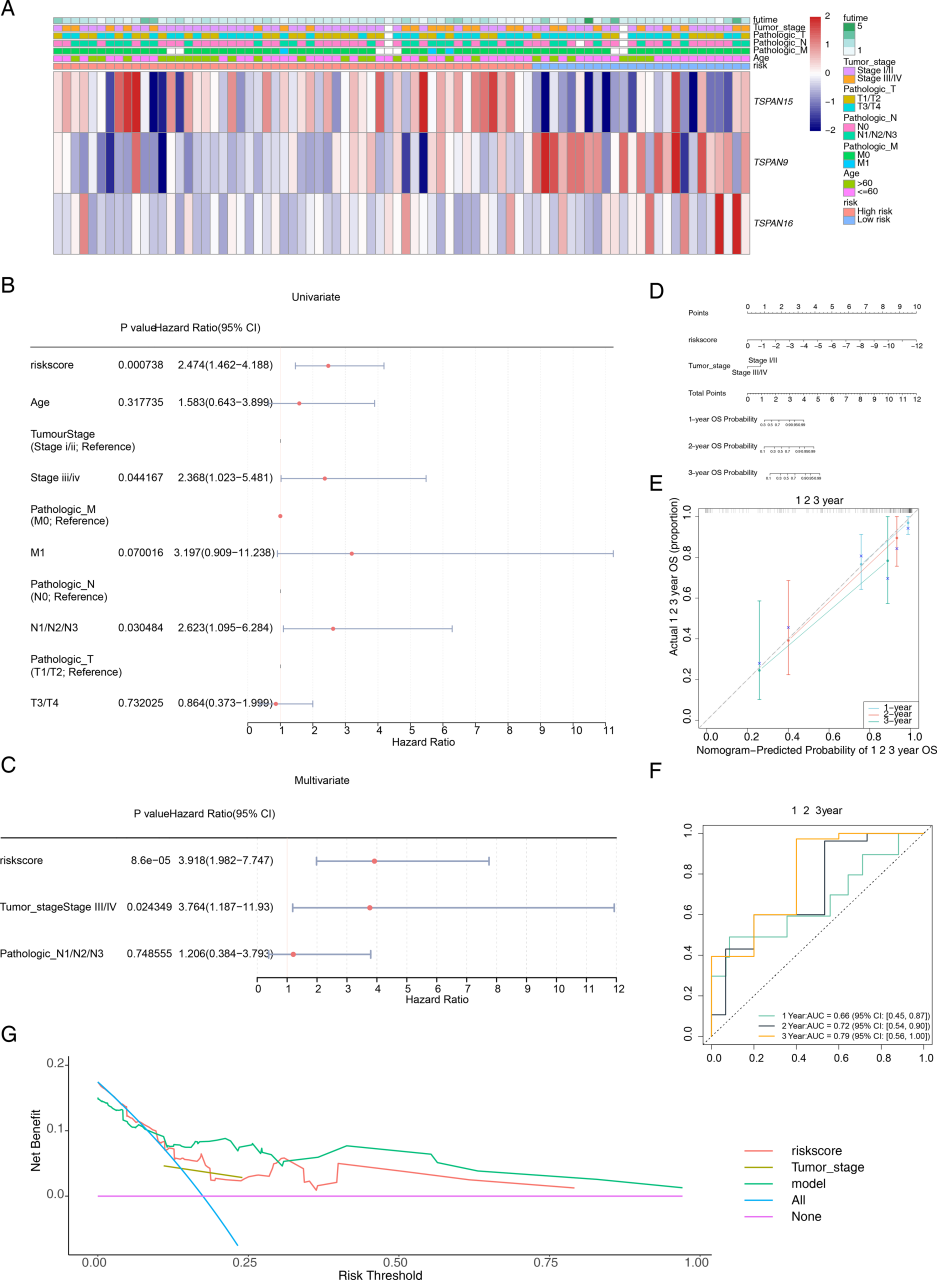
Construction of a prognostic model related to signature genes. **(A)** Distribution of clinicopathological features in the two risk groups. **(B)** Univariate Cox regression analyses of prognosis according to the risk score and clinical factors. Where *P* < 0.05 indicated that the clinical factor is associated with the patient’s survival prognosis. **(C)** Multivariate Cox regression analyses of prognosis according to the risk score and clinical factors. Where *P* < 0.05 indicated that the clinical factor is an independent prognostic factor. **(D)** The nomogram integrates the tumor stage and risk score for predicting the 1-, 2-, and 3-year OS of patients with ESCC. **(E)** The calibration curves and **(F)** ROC curves for the nomogram for evaluating the predictive value. **(G)** DCA of the nomogram model. The x-axis represents the risk threshold and the y-axis represents the net benefit. The purple line (“None”) indicates no intervention for any patients, and the blue line (“All”) indicates intervention for all patients. The red and brown lines represent the predictive effects of risk score and tumor stage, respectively, while the green line represents the predictive effect of the integrated nomogram.

Patients with varying clinical features require different therapeutic strategies and have different prognoses. We investigated the correlation between risk scores and clinical attributes. **Figure 5A** depicts the distribution of clinical traits and signature genes across various risk categories. To ascertain the efficacy of risk scores across various clinical subgroups as prognostic indicators, we conducted a K-M survival analysis and receiver ROC curve assessments for these subgroups. OS within high-risk group was considerably smaller than in low-risk group for M0 stage (*P* < 0.018), T3-T4 group (*P* < 0.017), and stage I-II group (*P* < 0.039). Additionally, AUC values at 1, 2, and 3 years exceeded 0.7 (**Supplementary Figure S2**). In the validation set GSE53622, high-risk patients aged 60 years or younger (*P* < 0.047), and those within T3-T4 group (*P* < 0.034) exhibited significantly shorter OS than low-risk patients. At the 1-year, 2-year, and 3-year marks, AUC values all surpassed 0.6 (**Supplementary Figure S3**). Although not statistically significant in other clinical subgroups, high-risk group patients typically exhibited a less favorable outcome compared to their low-risk counterparts. The research indicates that the risk score model exhibits enhanced precision in forecasting outcomes for patients in the T3-T4 stage, surpassing its predictive capabilities in other patient subsets. Building upon the clinical features and risk scores, we meticulously crafted a predictive nomogram for prognosis. The univariate Cox regression analyses exemplified that a risk score (*P* = 0.001, HR = 2.474, CI = 1.462 - 4.188), tumor stage (*P* = 0.044, HR = 2.368, CI = 1.023 - 5.481), and pathological N stage (*P* = 0.030, HR = 2.623, CI = 1.095 – 6.284) were associated with patients’ survival prognosis (**Figure 5B**). Among them, risk score, tumor stage and pathological N stage passed the PH assumption test. Risk score (*P* = 0.000086, HR = 3.918, CI = 1.982 - 7.747) and tumor stage (*P* = 0.024, HR = 3.764, CI = 1.187 - 11.93) were recognized as standalone predictive indicators (**Figure 5C**). Subsequently, an alignment chart incorporating risk score and tumor stage was developed to assess patient survival probabilities at 1–3 years (**Figure 5D**). Furthermore, these calibration curves closely matched the ideal curves (**Figure 5E**). AUC values for patients at 1–3 years consistently exceeded 0.6, with corresponding 95% CIs of 0.45–0.87, 0.54–0.90, and 0.56–1.00 (**Figure 5F**), demonstrating the model’s accuracy and validity. The Harrell’s C index was 0.741 (95% Cl: 0.647-0.835). The DCA curve verification showed that a net benefit value greater than 0 indicated a favorable predictive effect of the model (**Figure 5G**). The Brier score analysis demonstrated that the prediction error for 1-year OS was relatively low (0.10), indicating good accuracy at this time point. The prediction errors for 2-year OS (0.23) and 3-year OS (0.25) increased gradually, which is consistent with the general pattern observed in prognostic models, where predictive performance tends to decrease with longer follow-up.

### 3.4 Expression patterns of known ESCC prognostic genes across risk groups and clinical subgroups

**Figure 6:**
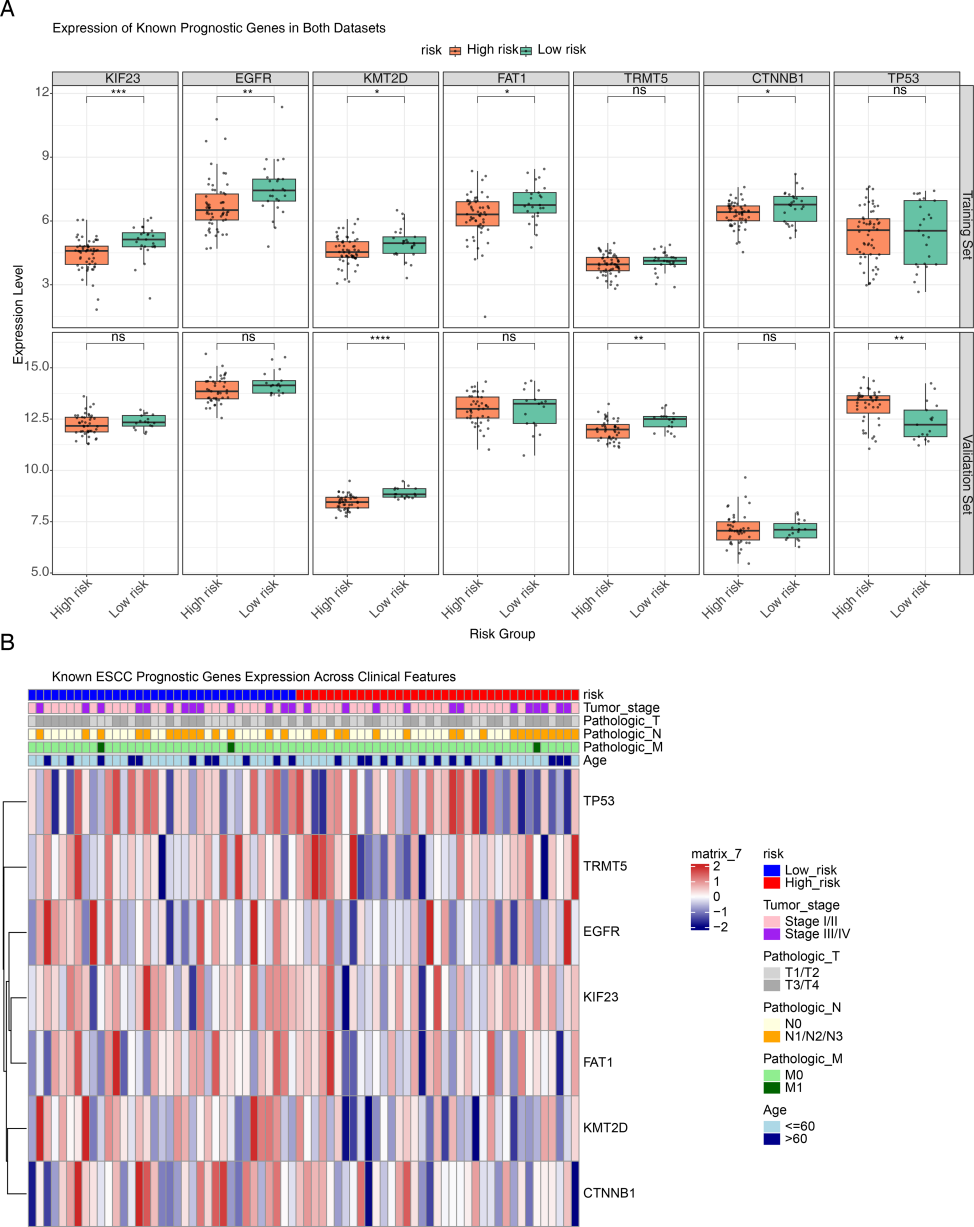
Expression patterns of known ESCC prognostic genes across risk groups and clinical strata. **(A)** Box plots illustrating the differential expression of ESCC prognosis-associated genes between high- and low-risk groups in the training and validation sets. The top row represents the training set, while the bottom row corresponds to the validation set. In each box plot, the x-axis indicates the high- and low-risk groups, and the y-axis represents the gene expression level. **(B)** Heatmap showing the distribution of seven prognostic genes across different clinical features. The top of the heatmap displays the risk groups along with major clinical features, while the heatmap body represents the relative expression levels of each gene in high-risk and low-risk groups. *P < 0.05, **P < 0.01, ***P < 0.001, ****P < 0.0001, *ns* no statistical significance.

To evaluate whether our *TM4SF*-based risk model captures established molecular phenotypes in ESCC, we analyzed the expression of seven previously validated prognostic genes. In the TCGA-ESCC cohort, several genes—including *KIF23*, *EGFR*, *KMT2D*, *FAT1*, and *CTNNB1*—were significantly upregulated in the low-risk group (*P* < 0.05). In the independent GSE53622 validation set, *KMT2D* and *TRMT5* showed marked upregulation in the low-risk group, whereas *TP53* was elevated in high-risk patients (*P* < 0.05). Notably, *KMT2D* was consistently downregulated in high-risk groups across both datasets. The concordant expression trends observed for most genes between the two independent cohorts support the robustness of our risk stratification approach (**Figure 6A**). Further analysis of gene expression across clinical strata revealed that genes such as *TP53* and *TRMT5* were generally expressed at higher levels in high-risk patients (**Figure 6B**). Importantly, this expression pattern correlated with adverse clinical features—including advanced tumor stage (III/IV), deeper invasion (T3/T4), and lymph node metastasis (N1/N2/N3)—suggesting that these molecular signatures align with an aggressive disease phenotype.

### 3.5 Differences in relevant pathways between risk groups

**Figure 7:**
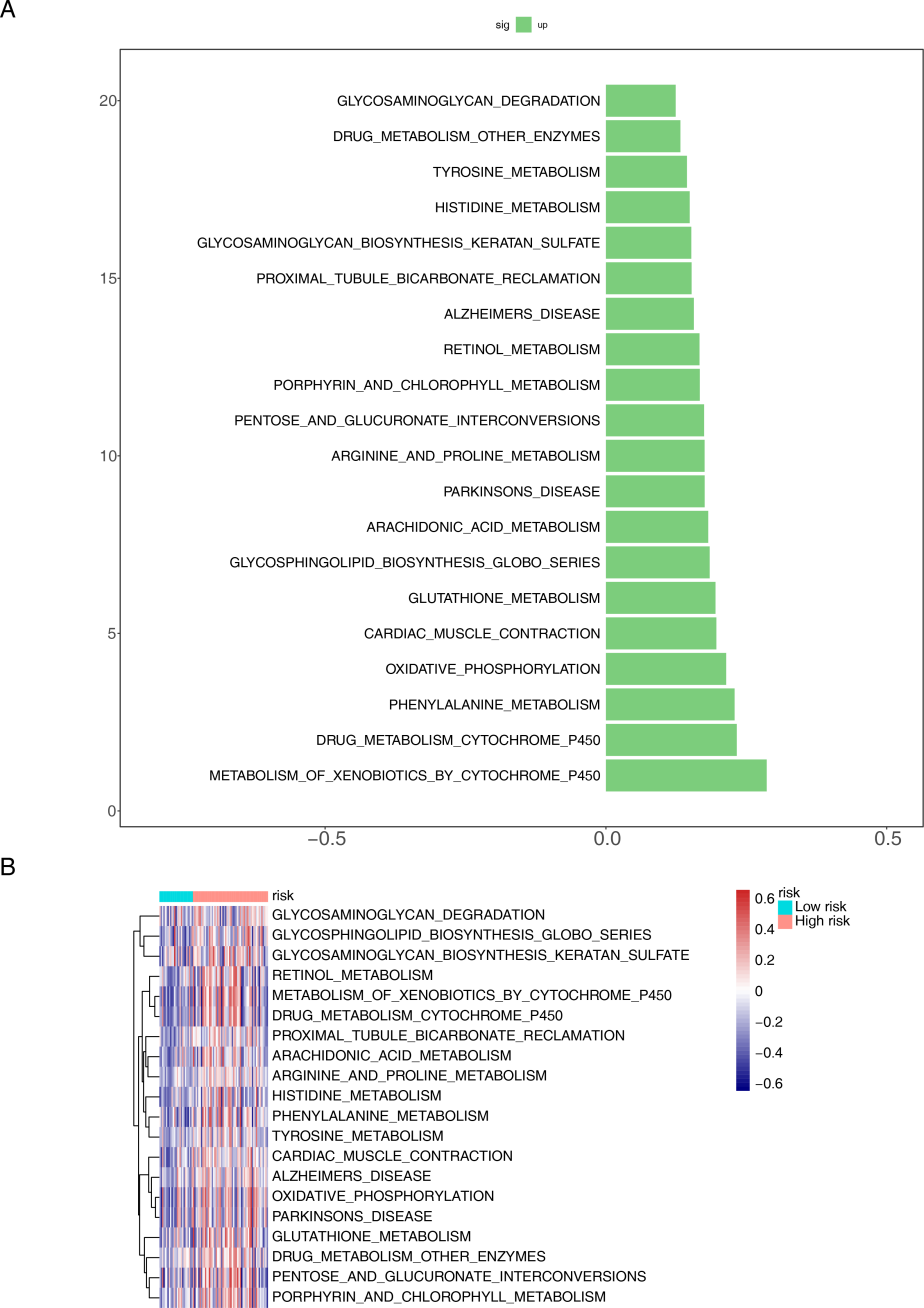
Functional enrichment analysis of differential genes between different risk groups. **(A)** Bar chart and **(B)** heat map for KEGG pathways.

We further examined pathway differences between distinct risk groups using GSVA. The examination uncovered notable activation across various pathways, such as the metabolism of xenobiotics by cytochrome P450, drug metabolism by cytochrome P450, phenylalanine metabolism, oxidative phosphorylation, and cardiac muscle contraction (**Figure 7A**). Furthermore, these pathways were highly prevalent within the high-risk cohort (**Figure 7B**).

### 3.6 Signature genes played an essential role in the immune microenvironment and better response to treatment in patients in high-risk group

**Figure 8:**
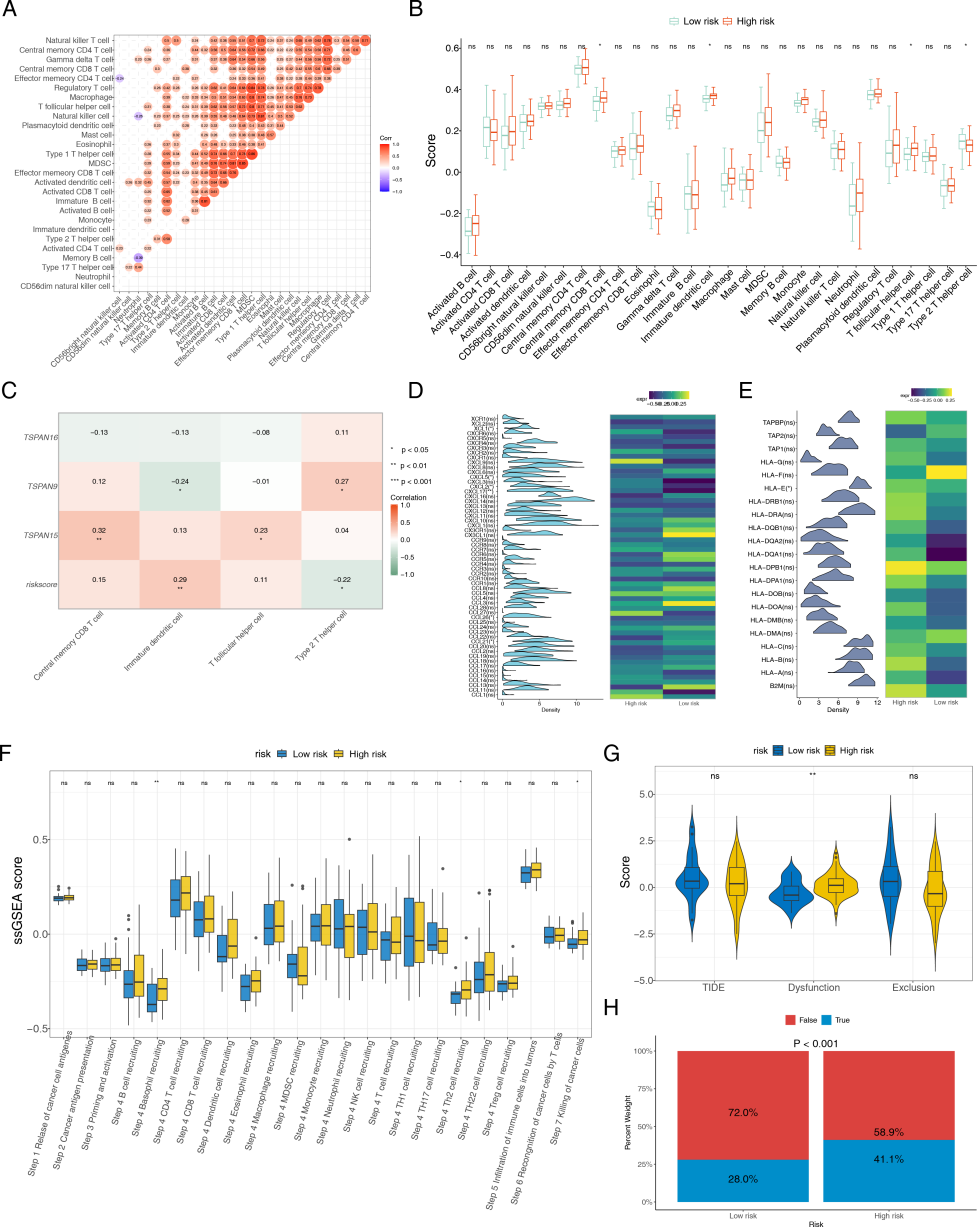
Association between the risk score and tumor microenvironment. **(A)** Correlation heatmap of 28 immune cells. **(B)** Distribution level of 28 immune cells in the high- and low-risk groups. B cell: B lymphocyte. T cell: T lymphocyte. CD4T: CD4^+^T lymphocyte. CD8T: CD8^+^T lymphocyte. MDCS: Myeloid-derived suppressor cell. **(C)** Heat map of the correlation between three signature genes and differentially expressed immune cells. Heat maps of the gene expression of **(D)** chemokines and their receptor-related genes, and **(E)***MHC* genes, between high- and low-risk groups. **(F)** The ssGSEA score of immune cycling processes in different risk groups. **(G)** Difference of TIDE, dysfunction and exclusion scores in different risk groups. **(H)** Comparisons of the proportions of no responders and responders to immunotherapy among high- and low-risk groups. *P < 0.05, **P < 0.01, ***P < 0.001, *ns* no statistical significance.

For further investigating the relationship among signature genes and immune microenvironment, we conducted immune infiltration analyses. Significant positive correlations were observed in the majority of the 28 immune cell types (**Figure 8A**). High-risk group showed elevated infiltration scores for central memory CD8 T cells, T follicular helper cells, and immature dendritic cells (DCs), whereas type 2 T helper (Th2) cells had reduced infiltration scores (**Figure 8B**). Additionally, a heatmap was employed to delineate the correlation matrix between triad of signature genes and the various subsets of differentially expressed immune cells. Although some prognostic genes are associated with immune cells (*P* < 0.05), the correlation is relatively low (cor < 0.5). *TSPAN9* and risk scores were significantly linked to type 2 T helper cells and immature DCs, while *TSPAN15* showed a marked positive correlation with T follicular helper cells (cor = 0.23) and central memory CD8 T cells (cor = 0.32) (**Figure 8C**). Additionally, *CCL26*, *CXCL2*, *CCL21*, *XCL1*, *CXCL5*, and *CXCL17* among chemokines and their receptor-related genes, and *HLA-E* among *MHC* genes were prominently expressed in different risk groups (**Figures 8D, E**). Furthermore, we collected gene sets representing immune cycling processes and analyzed them using ssGSEA. The data showed that immune cycling processes, specifically Step 4 basophil recruitment, Step 4 Th2 cell recruitment, and Step 7 killing of cancer cells, were substantially elevated within high-risk group (**Figure 8F**). To explore potential differences in immunotherapy response between risk groups, we applied the TIDE algorithm to TCGA datasets of ESCC patients. High-risk cohort exhibited elevated Dysfunction scores, indicating a propensity for impaired or abnormal tumor immunity (**Figure 8G**). Among the analyzed samples, a higher proportion of patients in the high-risk cohort were inferred to potentially show a positive response to immunotherapy (*P* < 0.001). (**Figure 8H**). These insights indicate that individuals within the high-risk category exhibit signs of immune system activation, implying a potentially enhanced response to immunotherapeutic treatments, although clinical data are still needed for verification. The results of the CIBERSORT analysis showed that most of the 22 types of immune cells exhibited a positive correlation with each other (**Supplementary Figure S4A**). When analyzing the immune cell infiltration levels between the high-risk and low-risk groups, significant differences were found only in the infiltration levels of two types of immune cells, CD4+ Tem and epithelial cells (*P* < 0.05) (**Supplementary Figure S4B**). The correlation analysis between characteristic genes and immune cells showed that *TSPAN9* had the strongest correlation with CD4+ Tem and epithelial cells, and both correlations were negative (**Supplementary Figure S4C**). This suggested that *TSPAN9* might be involved in the remodeling of the ESCC immune microenvironment by regulating the infiltration levels of these two types of immune cells.

### 3.7 Discussion of drug sensitivity and molecular regulation

**Figure 9:**
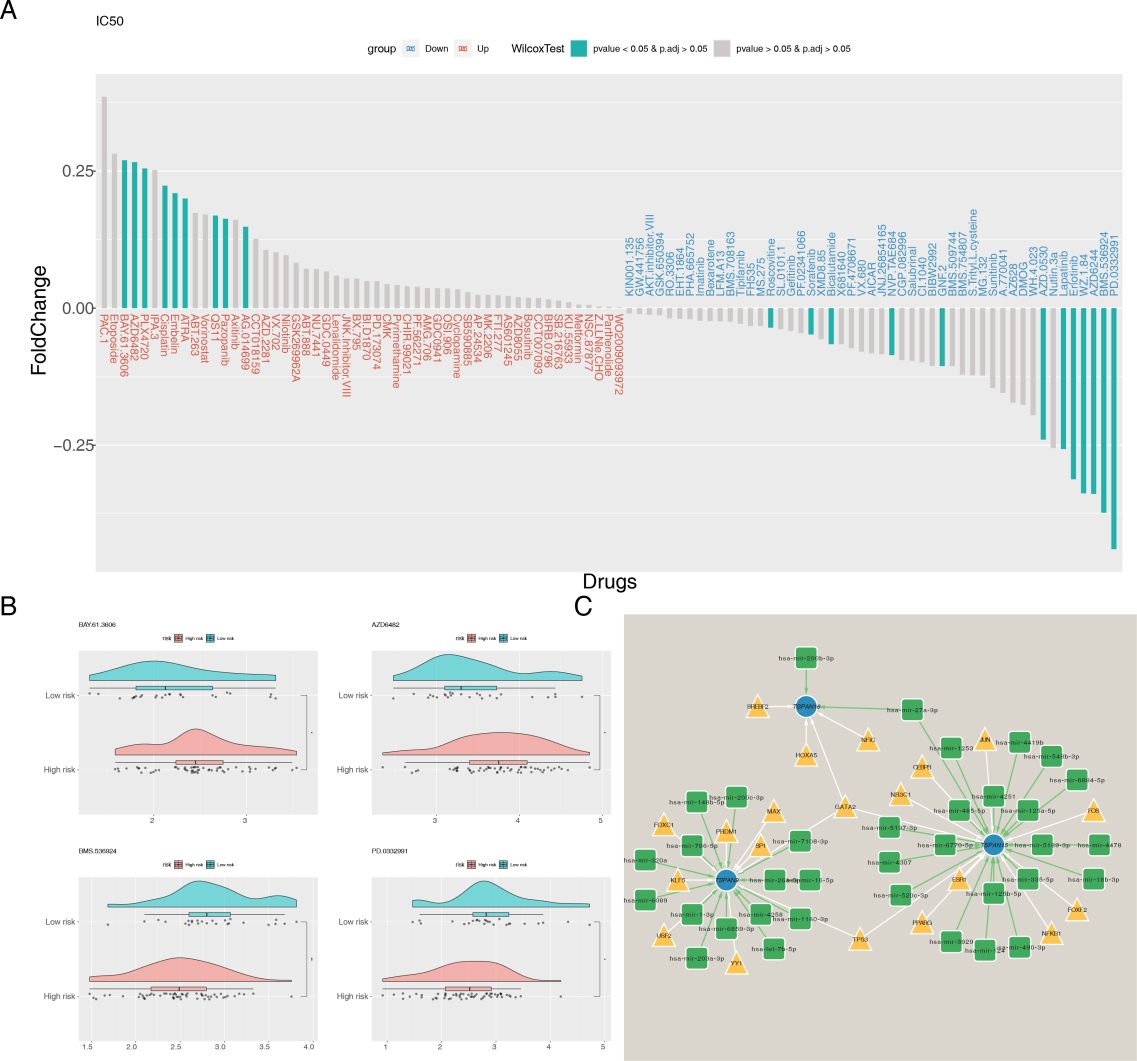
Chemotherapeutic drug sensitivity analysis and molecular network construction. **(A)** Differences in IC_50_ between risk groups for 138 common chemotherapeutic agents. **(B)** IC_50_ of BAY.61.3606, AZD6482, BMS.536924, and PD.0332991 in high- and low-risk groups. **(C)** The TF-mRNA-miRNA regulatory network. Blue nodes represent signature genes, yellow triangles represent TFs and green squares represent miRNAs. *P < 0.05, **P < 0.01.

To assess the susceptibility of individuals across various risk categories to various chemotherapeutic agents, we first calculated the IC_50_ values for 138 commonly used drugs. Wilcoxon test was used to evaluate differences between these risk groups. Among these, 21 drugs showed significant differences (**Figure 9A**), with BAY.61.3606, AZD6482, BMS.536924, and PD.0332991 showing the most notable difference (**Figure 9B**). Detailed information on 21 drugs can be found in **Supplementary Table S4**. The low-risk cohort exhibited enhanced responsiveness to BAY.61.3606 and AZD6482, whereas the high-risk cohort manifested a heightened sensitivity to BMS.536924 and PD.0332991. Based on these findings, individualized chemotherapy could be tailored for different risk populations. Additionally, TFs and miRNAs associated with the signature genes were predicted, and a TF-mRNA-miRNA network was constructed. All three signature genes were found to be regulated by TFs and miRNAs, with GATA2 regulating all three simultaneously (**Figure 9C**).

### 3.8 Expression validation of signature genes

**Figure 10:**
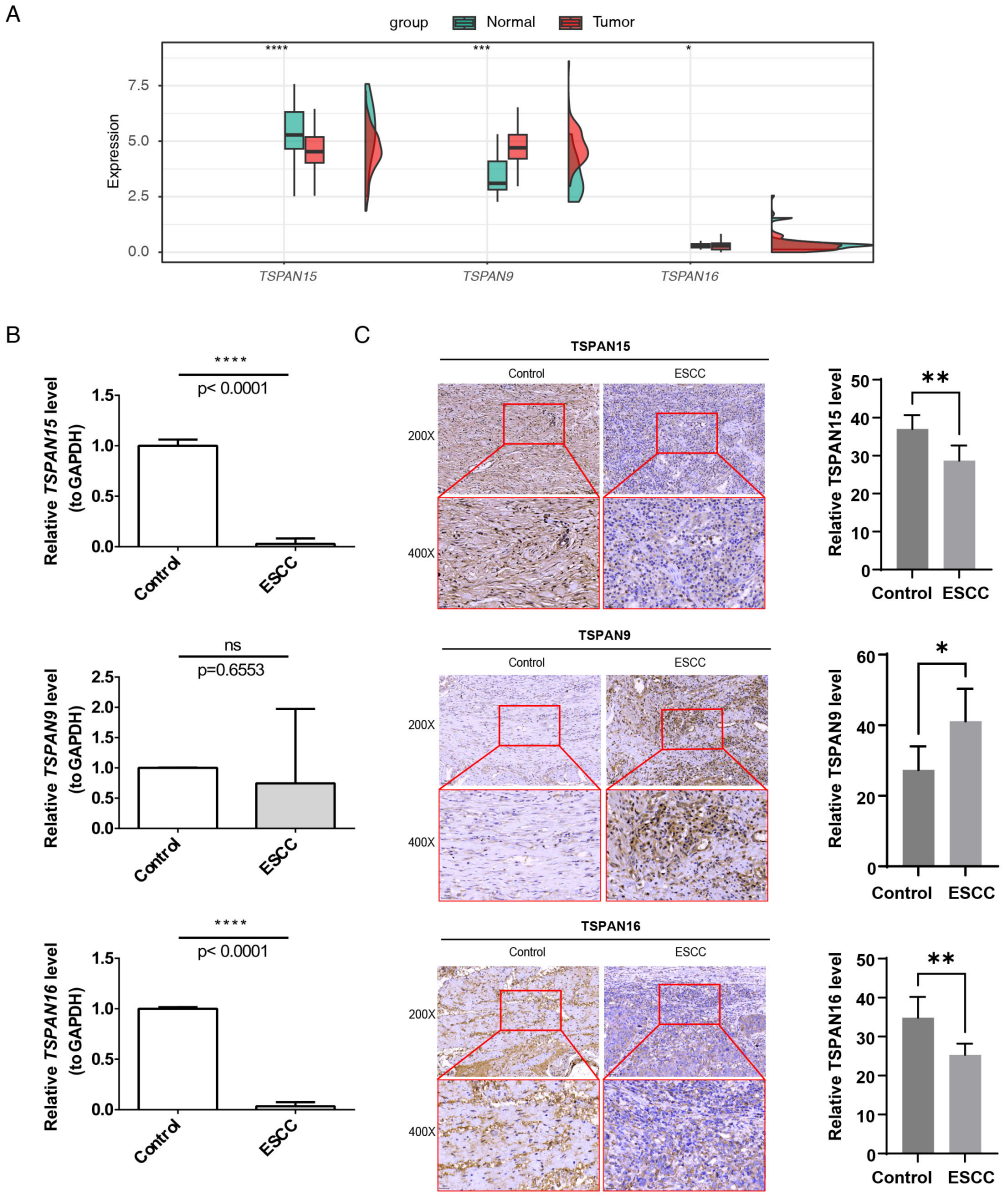
Expression validation of 3 signature genes. The mRNA expression levels of signature genes in **(A)** TCGA-ESCC and **(B)** patient tissue specimens. **(C)** Immunohistochemical detection of TSPAN15, TSPAN9, and TSPAN16 expression. Images captured at 200× magnification (scale bar = 50 µm) and 400× magnification (scale bar = 25 µm). *P < 0.05, **P < 0.01, ***P < 0.001, ****P < 0.0001, *ns*, no statistical significance.

In TCGA-ESCC, *TSPAN15* (*P* < 0.0001) and *TSPAN16* (*P* < 0.05) were considerably downregulated, while *TSPAN9* (*P* < 0.001) was notably upregulated (**Figure 10A**). Additionally, RT-qPCR results confirmed that *TSPAN15* (*P* < 0.0001) and *TSPAN16* (*P* < 0.0001) were also obviously downregulated within disease group, consistent with the expression trend observed in TCGA-ESCC (**Figure 10B**). Finally, IHC further validated our findings, showing that TSPAN15 (*P < 0.01*) and TSPAN16 (*P <* 0.01) were downregulated within disease group, while TSPAN9 (*P < 0.05*) was upregulated (**Figure 10C**).

## 4 Discussion

ESCC is a prevalent malignant tumor globally, characterized by poor prognosis, high recurrence, and high mortality rates (3). Despite the development of aggressive multimodal treatments over the past decades, treatment outcomes remain unsatisfactory (38, 39). Mounting research indicates that *TM4SF* is instrumental in progression, invasion, and metastasis of cancer cells (15–17). Nevertheless, the predictive significance of *TM4SF* in ESCC is yet to be fully elucidated. This investigation pinpointed *TSPAN15*, *TSPAN9*, and *TSPAN16* as signature genes associated with *TM4SF* in ESCC, and crafted an innovative predictive model capable of accurately forecasting the outcomes for patients with ESCC, while clarifying their significant roles in the ESCC tumor immune microenvironment.

This study identified 24 candidate DEGs correlated with *TM4SF* through differential analysis in ESCC. Enrichment analysis demonstrated that 24 prospective genes were intimately correlated with Notch signaling pathway, integrin binding, ion channel regulator activity, hematopoietic cell lineage, lysosome, and proteoglycans in cancer. Previous research has demonstrated that *TSPAN* proteins, like *CD9*, *CD81*, *CD151*, and *TM4SF1*, promote cancer metastasis by interacting with integrin α3β1 or α6, which aligns with our functional enrichment results (18, 40). A literature review revealed that *TSPANs* form dimers, such as *CD9-CD9* and *CD151-CD81*, serving as essential components in complexes involving *TSPANs* and other partners in cancer (17, 41). Homologous dimers are instrumental in the sphere of tumor biology, significantly impacting cellular processes including adhesion, migration, invasive capabilities, and signaling transduction (15, 42, 43). In summary, these findings highlight the relationship between ESCC and TRGs.

In this study, we identified *TSPAN15*, *TSPAN9*, and *TSPAN16* as signature genes associated with ESCC. The majority of research predominantly concentrates on the *TSPAN9* function in suppressing tumor development and progression, especially within the context of gastric cancer (44–46). Previous research indicates that *TSPAN9* has the potential to inhibit the migratory and invasive capabilities of gastric cancer SGC7901 cells via decreasing matrix metalloproteinase-9 (*MMP-9*) and urokinase-type plasminogen activator (*uPA*) secretion through extracellular signal-regulated kinases 1 and 2 (*ERK1/2*) pathway (44). Additionally, Elastic Microfibril Interface Located Protein 1 (*EMILIN1*) can synergistically inhibit gastric cancer cell invasion and metastasis through enhancing *TSPAN9* expression (45). A latest investigation indicates that *TSPAN9* boosts the resistance of gastric cancer cells to 5-FU by stimulating autophagy through the suppression of the *PI3K*/*AKT*/*mTOR* signaling pathway (46). Tan et al. (47) suggested that low expression of *TSPAN9* in hepatocellular carcinoma patients correlates in conjunction with an unfavorable prognosis. These findings align with our results regarding this prognostic impact of *TSPAN9* in ESCC. Conversely, mounting evidence denotes that *TSPAN15* serves as an oncogene, exerting an important influence on pathogenesis, progression, metastasis, and resistance to chemotherapy in cancer. Studies conducted both *in vitro* and *in vivo* have demonstrated that *TSPAN15* engages in a precise interaction with beta-transducin repeat-containing E3 ubiquitin-protein ligase (*BTRC*), facilitating the ubiquitination of phosphorylated IκBα (*p-IκBα*). This action primes *p-IκBα* for degradation by the proteasome. Consequently, this cascade results in the migration of nuclear factor-κB (*NF-κB*) to this cell nucleus, thereby contributing to an enhancement of metastatic potential in ESCC (48). *TSPAN15*, identified as a distinct binding affiliate of disintegrin and metalloproteinase 10 (*ADAM10*) (49), appears to participate in oncogenic mechanisms through an *ADAM10*-mediated pathway, as well as by stimulating *NF-κB* signaling (50–52). These investigations furnish further substantiation for our discovery that elevated expression levels of *TSPAN15* correlate with an unfavorable outcome. To date, the exploration of *TSPAN16* in the context of cancer remains scarce. Research indicates that *TSPAN16* is characteristically expressed at reduced levels in 33 varieties of cancer when contrasted with their corresponding normal tissue samples (10), which aligns with our findings. Our study identified *TSPAN16* as being regulated by hsa-miR-200b-3p, a microRNA with well-documented roles in cancer initiation and progression. For example, increasing hsa-miR-200b-3p expression may help restore the suppressive influence of Noxa on gastric carcinoma cell proliferation (53). Furthermore, the lengthy non-coding RNA known as X-inactive specific transcript (*XIST*) functions as a molecular absorbent for miR-200b-3p, thereby regulating the expression of zinc finger E-box binding homeobox (*ZEB*) 1/2 and consequently stimulating the proliferation, migration, and invasive capabilities in hepatocellular carcinoma (54). However, these specific regulatory interactions between *TSPAN16* and hsa-miR-200b-3p in ESCC warrant further investigation. Our findings are the first to suggest that *TSPAN16* significantly impacts prognostic prediction in ESCC patients, offering a new perspective for future research. To further substantiate the robustness of our bioinformatics findings, we conducted expression validation at both the transcript and protein levels. Specifically, mRNA expression of the signature genes was confirmed using TCGA-ESCC data and RT-qPCR, while protein expression was validated by IHC in patient tissue samples. These complementary approaches provide consistent evidence supporting the reliability of our analysis.

This study developed an innovative risk model to accurately predict ESCC prognosis. Several prognostic biomarkers and predictive models have already been proposed for ESCC. A recent study established a novel risk model based on cancer-associated fibroblasts, achieving satisfactory AUC values. This model can also effectively predict OS and immunotherapy outcomes in ESCC patients (55). Prior research has indicated that pyroptosis plays a significant role in initiation and advancement of diverse types of cancers. Zhang et al. evolved a risk model for ESCC using four pyroptosis-related genes, which revealed poorer survival outcomes within the high-risk group (56). This research marks a pioneering effort in exploring this prognostic predictive power of a risk model founded on TRGs in ESCC patients. The model has showcased remarkable coherence and a potent predictive capacity concerning the outcomes for ESCC patients. To validate the broad applicability of the model, we employed an independent validation dataset, GSE53622. The results showed that the AUC values for 1-, 2-, and 3-year survival predictions were 0.739, 0.666, and 0.640, respectively. Although a moderate decline was observed over time, the values consistently remained above 0.6, indicating stable predictive performance. This gradual decrease in predictive efficiency over time is a common phenomenon observed in prognostic models, primarily due to the accumulation of unmeasured clinical events and therapeutic interventions that may dilute the initial prognostic signal (57). In addition, tumor heterogeneity and clonal evolution dynamically alter the molecular landscape, thereby weakening the predictive power of baseline gene expression features for long-term outcomes (58). Similar time-dependent declines in AUC have also been documented in other ESCC prognostic models (9, 56). Despite limitations such as the sample size of the validation cohort, our model maintained robust performance (AUC > 0.6 at all-time points), supporting its reliable clinical applicability for short- to medium-term survival prediction. To ascertain the model’s efficacy more comprehensively, we carried out an exhaustive examination of its efficacy among different patient demographics. We found that combining the risk score with tumor stage significantly improved the accuracy and reliability of survival predictions for ESCC patients. Notably, in the T3 and T4 stage patient groups, the risk score demonstrated a significant prognostic benefit over other subgroups, consistently achieving AUC values above 0.6 for 1-year, 2-year, and 3-year predictions. This not only further validates the model’s clinical application potential but also underscores its significance and value in clinical practice.

We also compared our TRGs-based risk model with seven well-established ESCC prognostic genes, including *TP53*, *TRMT5*, *EGFR*, *KIF23*, *FAT1*, *KMT2D*, and *CTNNB1* (27–30). This comparison showed that the expression patterns of these genes were consistent with our model in both high- and low-risk groups across two independent datasets (TCGA-ESCC and GSE53622). These consistent results across multiple datasets reinforce the reliability of our TRGs-based model, supporting its potential as a complementary tool alongside traditional prognostic markers in ESCC.

Over the last decade, cancer immunotherapy has emerged as a powerful treatment modality, heavily dependent on understanding the immune landscape within tumor microenvironments (59). This study examined the immune landscape of ESCC using a *TM4SF*-related risk signature. Low-risk group showed significantly higher levels of Th2 cell infiltration. Research by Schreiber et al. (60) indicated that Th2 cell-mediated type 2 immunity may enhance anti-tumor immune responses. Mattes et al. (61) revealed that tumor-bearing mice receiving ovalbumin-specific Th2 cells effectively cleared lung and visceral melanoma metastases through M2 macrophage recruitment. Peng and colleagues (62) discovered a significant positive association between the levels of Th2 cells and OS in ESCC patients who did not undergo postoperative chemotherapy, indicating its promising role as a prognostic indicator. Interestingly, high-risk group showed elevated levels of central memory CD8+ T cells, T follicular helper cells, and immature DCs. DCs within the tumor microenvironment display dual functionality, with research indicating that their pro-oncogenic effects result from activating regulatory T cells to inhibit anti-tumor immune responses (63). As a result, DC recruitment in cancer correlates with poor prognosis (64, 65), potentially explaining their high expression in high-risk groups. Furthermore, our research has uncovered a crucial friendship among risk scores and factors such as chemokines, chemokine receptors, *MHC* genes, and immune cycle processes in ESCC. Notably, *HLA-E* is the only *MHC* component that expression levels notably vary between high-risk and low-risk populations, exhibiting reduced regulation within the high-risk cohort. This suggests that its lower expression may be associated with poor prognosis in ESCC patients. Research by Xu et al. (66) demonstrated that patients with elevated levels of *HLA-E* immunostaining experienced significantly longer OS compared to those with lower levels, supporting our hypothesis. In line with this, increased *HLA-E* expression was linked to extended survival for several human tumors, including cervical adenocarcinomas (67) and glioblastomas (68). These findings suggest that risk score metrics could be critically involved in modulating the immune responsiveness of tumor cells to immunotherapeutic interventions. TIDE results provide preliminary clues that high-risk patients may exhibit relatively better responses to immunotherapy compared with low-risk patients, which is consistent with our initial hypothesis. However, it should be emphasized that this inference is based solely on computational simulation using the TIDE algorithm, and its clinical relevance must be further validated through prospective clinical data.

Our immune infiltration analysis using the CIBERSORT algorithm revealed distinct immune cell subsets between high- and low-risk groups. Specifically, we observed statistically significant differences in the infiltration levels of CD4+ Tem and epithelial cells. In contrast, the infiltration levels of CD8+ Tem did not show statistically significant differences. Although CD8+ Tem, as a critical effector population in anti-tumor immunity, did not show a significant difference in our cohort, their role in ESCC cannot be overlooked. The presence of effector memory T cells (including CD4+ Tem and CD8+ Tem) is essential for maintaining long-term anti-tumor immune responses (69, 70). The significant changes in CD4+ Tem can provide critical help for the activation and function of CD8+ T cells, while the lack of significant changes in CD8+ Tem might indicate a more complex qualitative dysfunction in the T-cell compartment of high-risk ESCC (71). For example, CD8+ Tem cells in the high-risk tumor microenvironment may be in a state of functional exhaustion or impairment, which might not be reflected merely by their numbers (59, 72). This concept is supported by other cancer studies, which indicate that the functional status of T cells often predicts prognosis and treatment response more accurately than their absolute numbers (73). Therefore, while our quantitative analysis highlighted CD4+ Tem as a key differential subset, future studies should incorporate functional markers such as Programmed Cell Death Protein 1 (*PD-1*), T Cell Immunoglobulin and Mucin-Domain Containing-3 (*TIM-3*), and Granzyme B (*GZMB*) to explore the roles of CD4+ Tem and CD8+ Tem in the *TM4SF*-defined ESCC subtype (73–75), which may reveal immune escape mechanisms beyond changes in cell infiltration numbers.

Additionally, we further investigated variations in responsiveness to chemotherapeutic agents among the high- and low-risk groups. Our comprehensive examination has uncovered that BAY.61.3606 and AZD6482 showed greater efficacy within low-risk group, while BMS.536924 and PD.0332991 were more effective within high-risk group. BMS-536924 has been reported to effectively inhibit an activation of *Akt* and mitogen-activated protein kinases (*MAPK*), thereby enhancing 5‐fluorouracil (5‐FU)-induced apoptosis in a manner proportional to the dose administered, along with exhibiting anti-neoplastic effects in esophageal cancer cells (76). Similarly, PD‐0332991, a potent inhibitor of cyclin D1‐cyclin‐dependent kinase 4/6 (*CDK4/6*), has been shown curtail cell proliferation, induce apoptosis and senescence, and suppress migration, invasion, and metastasis in ESCC. Additionally, it has been found to enhance the effectiveness of 5‐FU and cisplatin in ESCC cells (77). Currently, no studies have reported the role of BAY.61.3606 and AZD6482 in ESCC. However, previous research has demonstrated that these compounds hold promising potential in cancer therapy—not only by exerting clear anti-neoplastic effects to suppress cancer cell growth, but also by significantly enhancing the sensitivity of cancer cells to targeted molecular therapies, thereby providing important rationale for their potential application in oncology (78). Nevertheless, additional investigation is requisite to ascertain their precise functions in ESCC. These findings could guide personalized chemotherapy and targeted therapy approaches.

Nevertheless, these findings should be interpreted with caution. The drug sensitivity predictions in this study were generated using the pRRophetic algorithm, which is based on Genomics of Drug Sensitivity in Cancer (GDSC) cell-line data. *In vitro* cell-line models differ substantially from the *in vivo* tumor microenvironment (TME) and thus may not fully capture the actual therapeutic responses in patients. Conventional two-dimensional cell lines, typically derived from monoclonal cultures, only reflect basic biological behaviors of tumor cells but fail to reproduce the complex interactions among tumor cells, stromal cells, immune cells, and extracellular matrix components (59, 79, 80). Consistently, our functional enrichment analysis revealed pathways related to integrin binding and the Notch signaling pathway, both of which involve stromal–tumor interactions that are absent in monoculture systems. Moreover, ESCC is characterized by a unique immunosuppressive landscape and dynamic immune-cell equilibrium, which cannot be replicated in cell-line systems. Recently, ESCC organoid models have emerged as more physiologically relevant preclinical platforms that preserve tissue architecture and tumor heterogeneity, providing an opportunity to bridge the gap between cell-line predictions and patient biology (81). Therefore, while our pRRophetic results provide useful preliminary insights, further validation in ESCC organoid models or *in vivo* animal models will be necessary to confirm the therapeutic implications.

To enhance comprehension of the relationships and potential regulatory mechanisms among signature genes, we established regulatory loops involving TFs, mRNAs, and miRNAs. Our analysis revealed that *GATA2* can target *TSPAN15*, *TSPAN16*, and *TSPAN9*. *GATA2*, a member of the *GATA* family of transcription factors (*GATA1*–*GATA6*), binds to the “*GATA*” DNA motif via two zinc-finger domains (82). Recent research has indicated that *GATA2* fulfills a function in transcriptional regulation of specific ESCC target genes, although the precise mechanisms remain incompletely understood (83). Thus, it is hypothesized that *GATA2* may contribute to tumorigenesis and progression by regulating the transcription of signature genes (*TSPAN15*, *TSPAN16*, and *TSPAN9*). Therefore, additional investigation is essential to delve into this conjecture.

This study has several limitations, which should be noted. This represented a backward-looking examination of information culled from publicly accessible databases; therefore, the potential for selection and confounding biases was unavoidable. Moreover, no prospective validation or cross-validation in independent clinical cohorts or other external datasets was performed, which may limit the robustness of the conclusions. Finally, while the training cohort from TCGA-ESCC was composed predominantly of non-Asian patients, the validation cohort (GSE53622) consisted entirely of Asian patients. Considering the known differences in etiology, genetic background, and molecular characteristics of ESCC across ethnic groups, the global applicability of our model remains uncertain, and its predictive performance in non-Asian populations requires further confirmation. Furthermore, the relatively small sample size of the validation cohort and the imbalance between high- and low-risk groups defined by the optimal cutoff may have affected statistical power. As real-world clinical prognostic and treatment response data were not incorporated, the clinical utility and predictive performance of the model cannot yet be fully established. These factors may affect the robustness and broad applicability of our findings. At the experimental and mechanistic level, this study only validated molecular expression using RT-qPCR and IHC, without clinical correlation analyses or functional experiments, leaving the mechanistic interpretation incomplete. Moreover, the TIDE algorithm has been validated primarily in melanoma and non-small-cell lung cancer; given the distinct tumor microenvironment of ESCC, its predictive results in this context remain uncertain. To address the above limitations and to further validate and extend our findings, future work will focus on three major directions: First, conducting large-scale prospective studies incorporating multi-center and multi-ethnic ESCC cohorts (including both Asian and non-Asian populations) with comprehensive real-world clinical data, including long-term follow-up and treatment response records, to enhance the accuracy, reliability, and generalizability of the conclusions. Second, performing systematic functional experiments to elucidate the roles of the signature genes in ESCC, characterize the downstream regulatory networks of key transcription factors such as *GATA2*, and identify their molecular targets in ESCC pathogenesis, while simultaneously leveraging clinical datasets such as KEYNOTE-181 (84) and KEYNOTE-590 (85) to validate and calibrate the TIDE algorithm and to identify ESCC-specific immunological biomarkers. Third, based on refined cohort data and mechanistic insights, further optimizing the prognostic model to better meet clinical needs and exploring the potential of the identified molecules as diagnostic biomarkers or therapeutic targets, thereby providing new strategies for precision diagnosis and treatment.

## 5 Conclusions

For the first time, we identified signature genes for ESCC associated with *TM4SF*, including *TSPAN15*, *TSPAN9*, and *TSPAN16*, and constructed a risk model that effectively predicts ESCC prognosis. This risk model demonstrates remarkable performance in independently evaluating ESCC prognosis and offers potential guidance for tumor-targeted therapies.

## Data Availability

The datasets presented in this study can be found in online repositories. The names of the repository/repositories and accession number(s) can be found in the article/**Supplementary Material**.
