# Supplementary material for: Construction and validation of a transmembrane 4 superfamily-related genes prognostic model for esophageal squamous cell carcinoma
Source: Front Oncol. 2025 Nov 19;15:1580199. doi: 10.3389/fonc.2025.1580199 (PMC12672243; doi:10.3389/fonc.2025.1580199)
Supplement: Supplementary file 1 [file DataSheet1.zip › Supplementary Material/Supplementary Material.DOCX]

Supplementary Material

# Supplementary Tables

## Supplementary Table S1

Specific conditions for amplification reactions.

| **Stage** | **Temperature** | **Time/Number of times** |
| --- | --- | --- |
| Permutability | 95℃ | 1min |
| Degenerative | 95℃ | 20s |
| Annealing | 55℃ | 20s |
| Cycles | - | 40 times |
| Extension | 72℃ | 30s |

## Supplementary Table S2. The ΔΔCt value of the qPCR experiment

*Note: Due to the size and detail of this dataset,* ***Supplementary Table S2*** *is provided separately as an Excel file (.xlsx).*

## Supplementary Table S3

Primers used in this study.

| **Primer** | **Sequences** |
| --- | --- |
| *TSPAN15* F | ACCGTGTTCTGGACCATTGACT |
| *TSPAN15* R | TGCACACTGAAACGCTCCTT |
| *TSPAN9* F | ACCCAAAGGCAGCAAGTACG |
| *TSPAN9* R | GCAGACGATGCCTCCAATTC |
| *TSPAN16* F | AGGCACGCTCTTGTTTGTTG |
| *TSPAN16* R | TTCCTGGCAACTTCTGGTGG |
| GAPDH F | CGAAGGTGGAGTCAACGGATTT |
| GAPDH R | ATGGGTGGAATCATATTGGAAC |

## Supplementary Table S4. Detailed information table of 21 kinds of drugs

*Note: Due to the size and detail of this dataset,* ***Supplementary Table S4*** *is provided separately as an Excel file (.xlsx).*

# Supplementary Figures

## Supplementary Figure S1


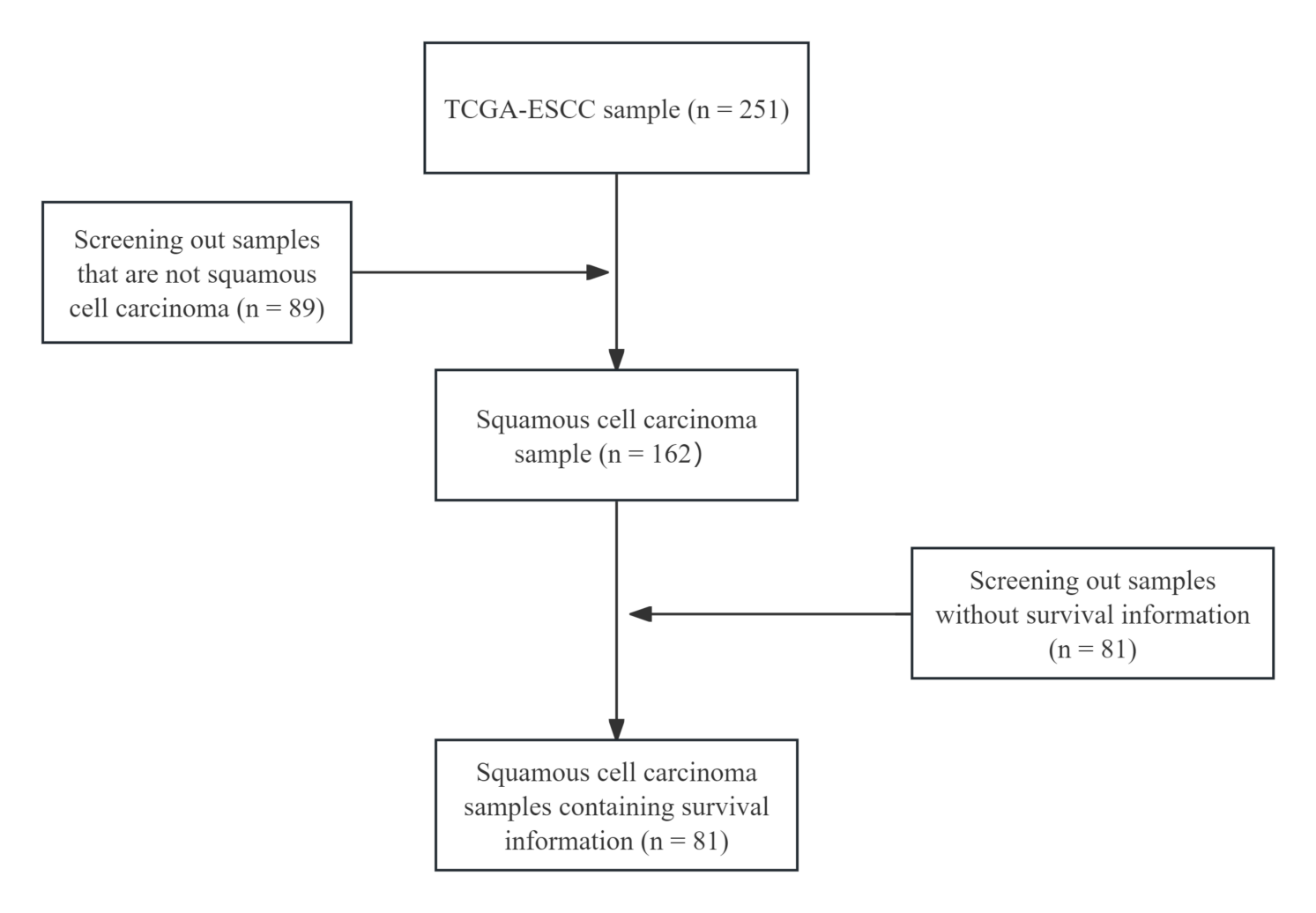


**Supplementary Figure S1.** CONSORT-style flow diagram of TCGA-ESCC sample selection, showing how 251 initial cases were filtered to obtain the final cohort of 81 ESCC samples with complete survival data.

## Supplementary Figure S2

**
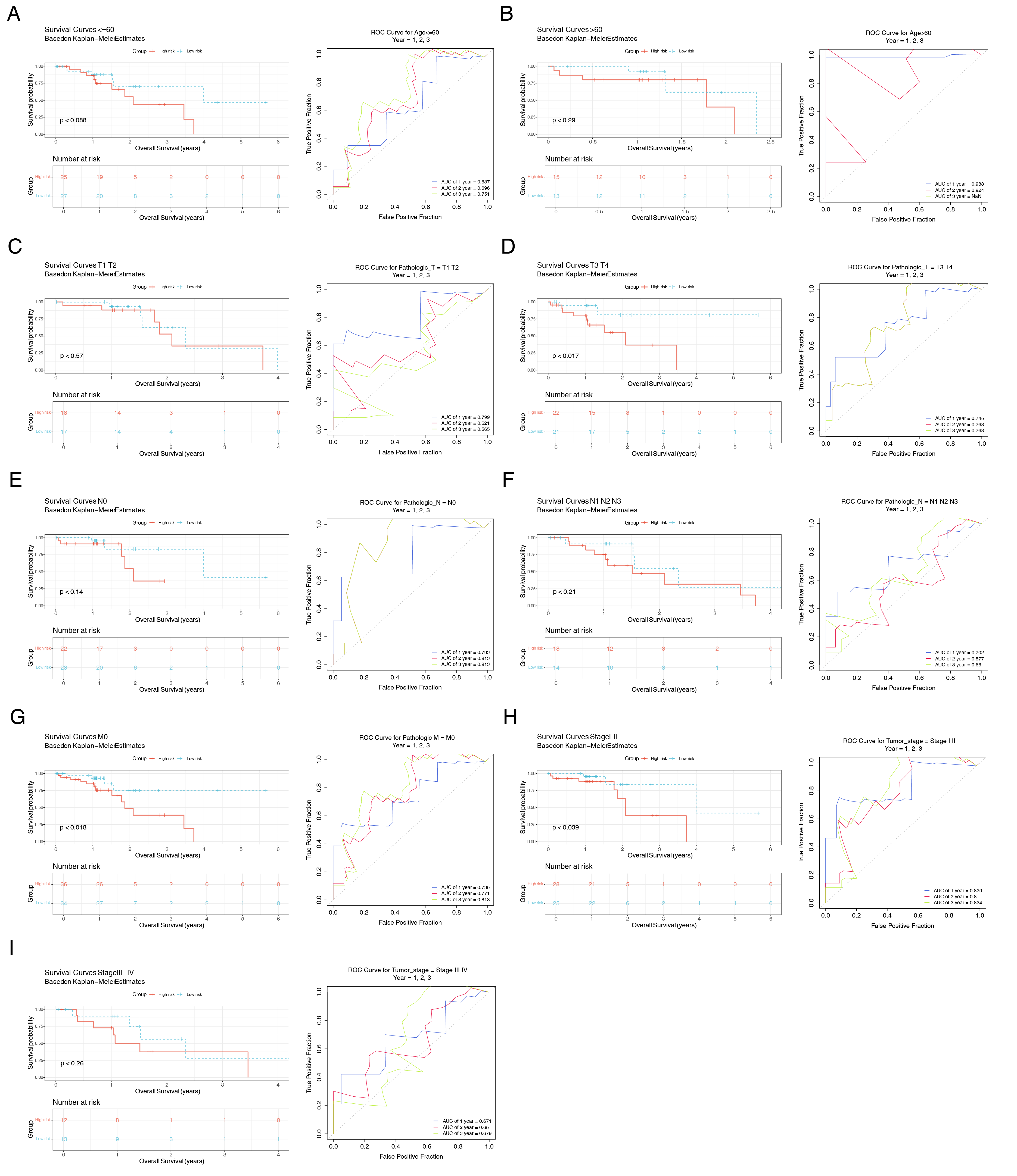
Supplementary Figure S2.** K-M survival analysis with ROC analysis of risk scores for different clinical subgroups (training set TCGA-ESCC). **(A-B)** Age. **(C-D)** T stage. **(E-F)** N stage. **(G)** M stage. **(H-I)** Tumor stage.

## Supplementary Figure S3


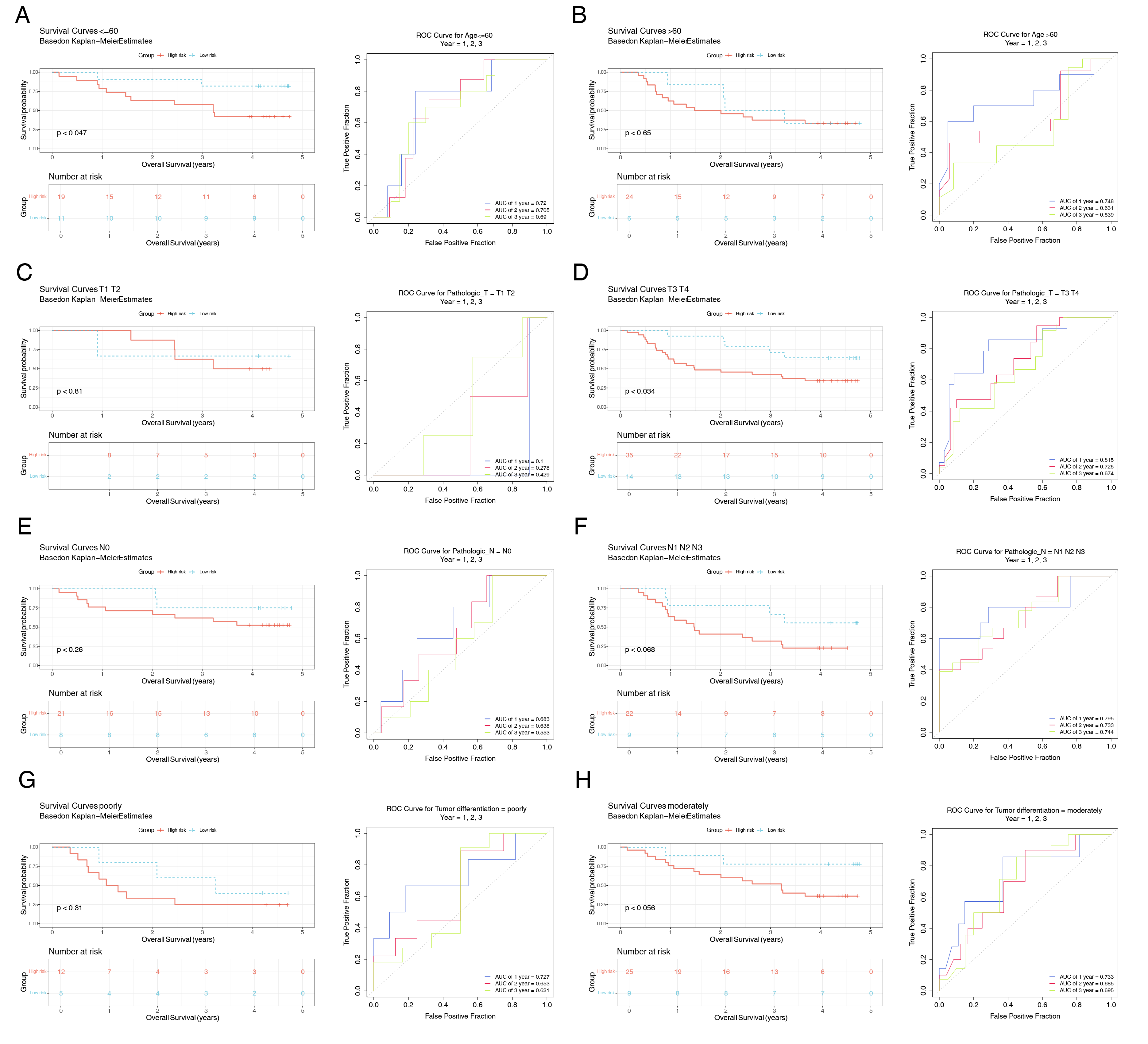


**Supplementary Figure S3.** K-M survival analysis with ROC analysis of risk scores for different clinical subgroups (validation set GSE53622). **(A-B)** Age. **(C-D)** T stage. **(E-F)** N stage. **(G-H)** Differentiation degree.

## Supplementary Figure S4


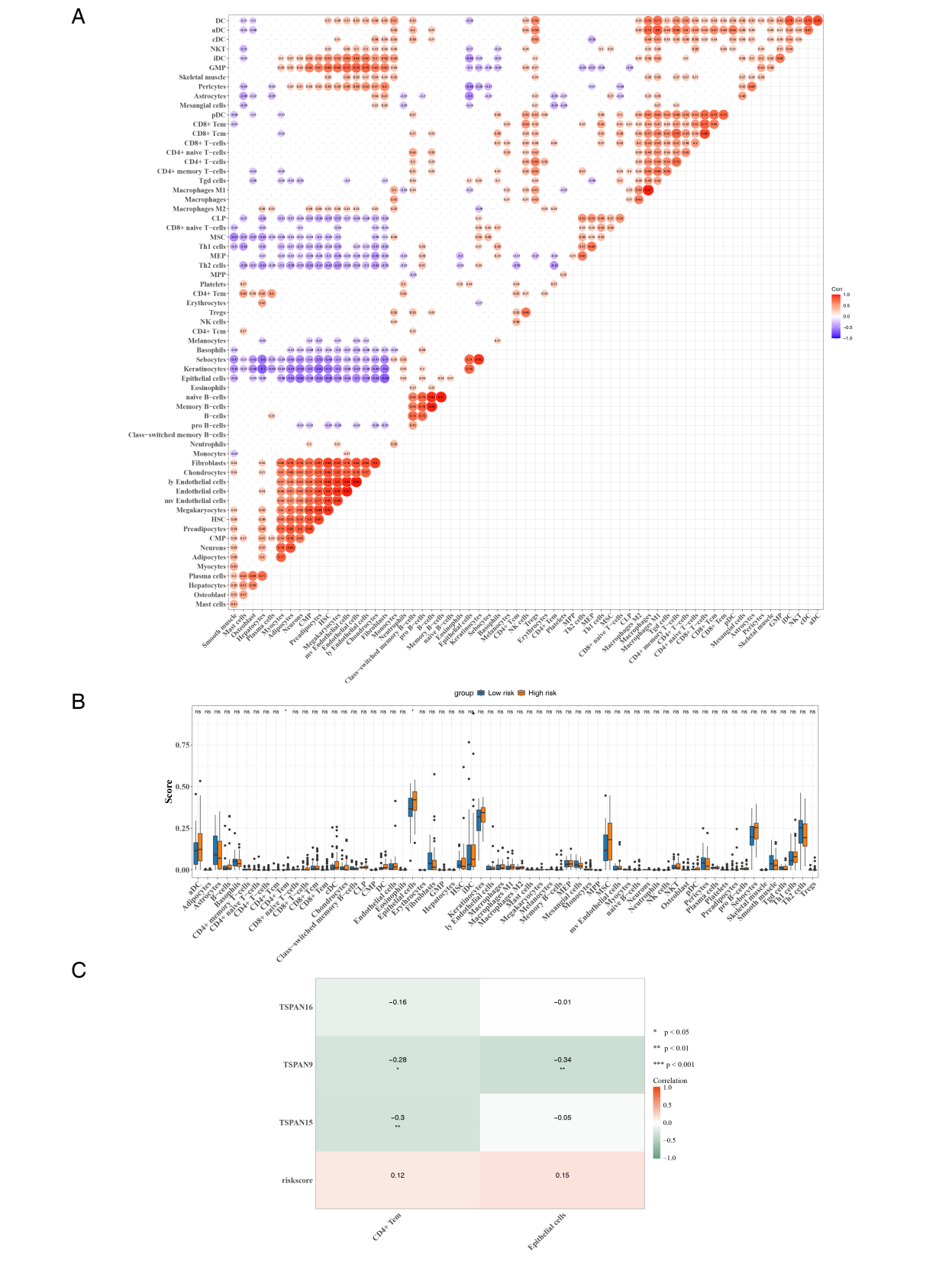
**Supplementary Figure S4.** Immune infiltration analysis using the CIBERSORT algorithm. (A) Correlation heatmap of 22 immune cell types. (B) Boxplot showing the relative abundance of 22 immune cell types between groups. (C) Heatmap illustrating the correlations between signature genes and immune cell infiltration.
