# Supplementary figures and images for: Construction and validation of a transmembrane 4 superfamily-related genes prognostic model for esophageal squamous cell carcinoma
Source: Front Oncol. 2025 Nov 19;15:1580199. doi: 10.3389/fonc.2025.1580199 (PMC12672243; doi:10.3389/fonc.2025.1580199)

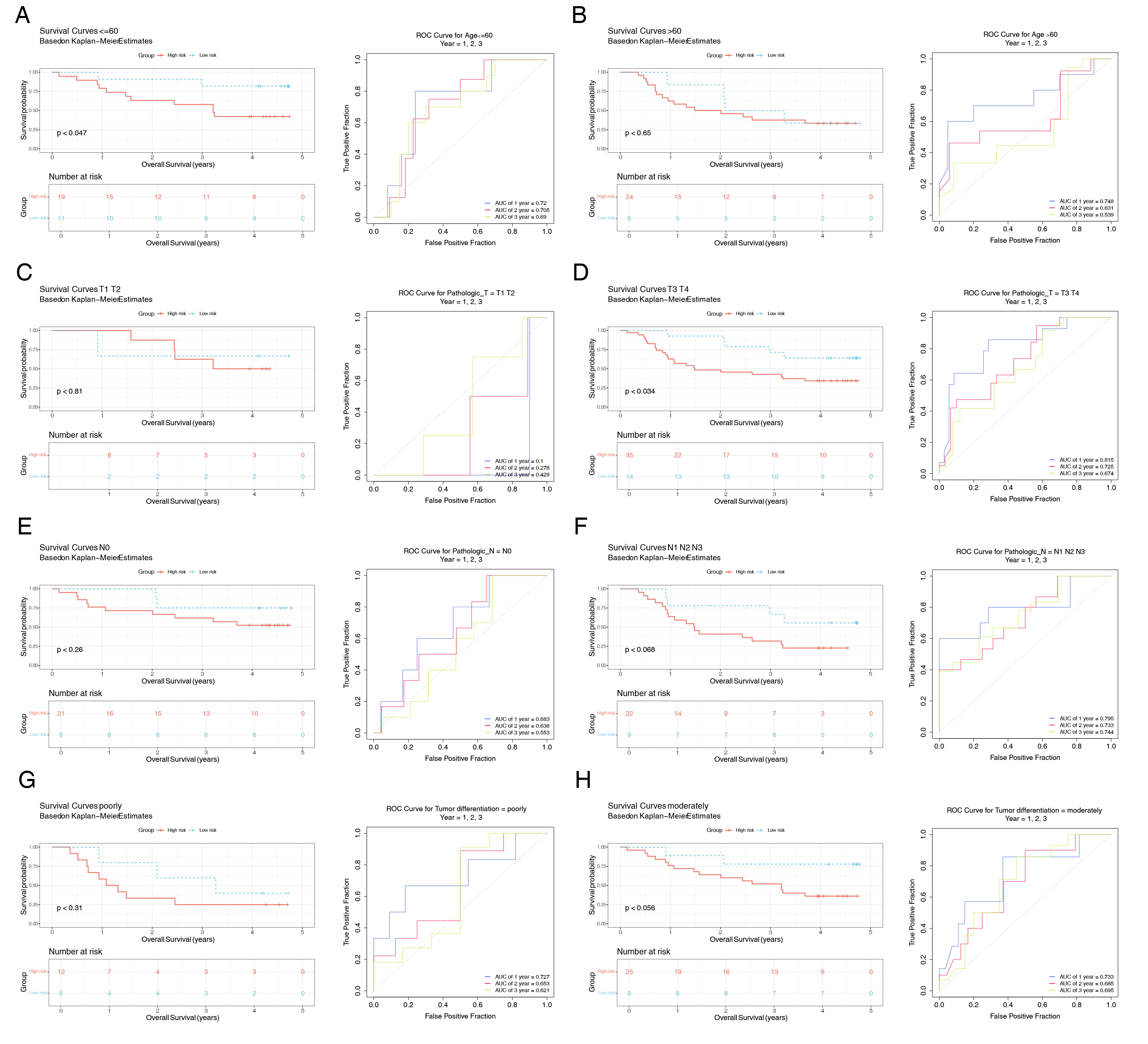

Supplement: Supplementary file 1 [file DataSheet1.zip › Supplementary Material/Supplementary Figure S3 .TIF]

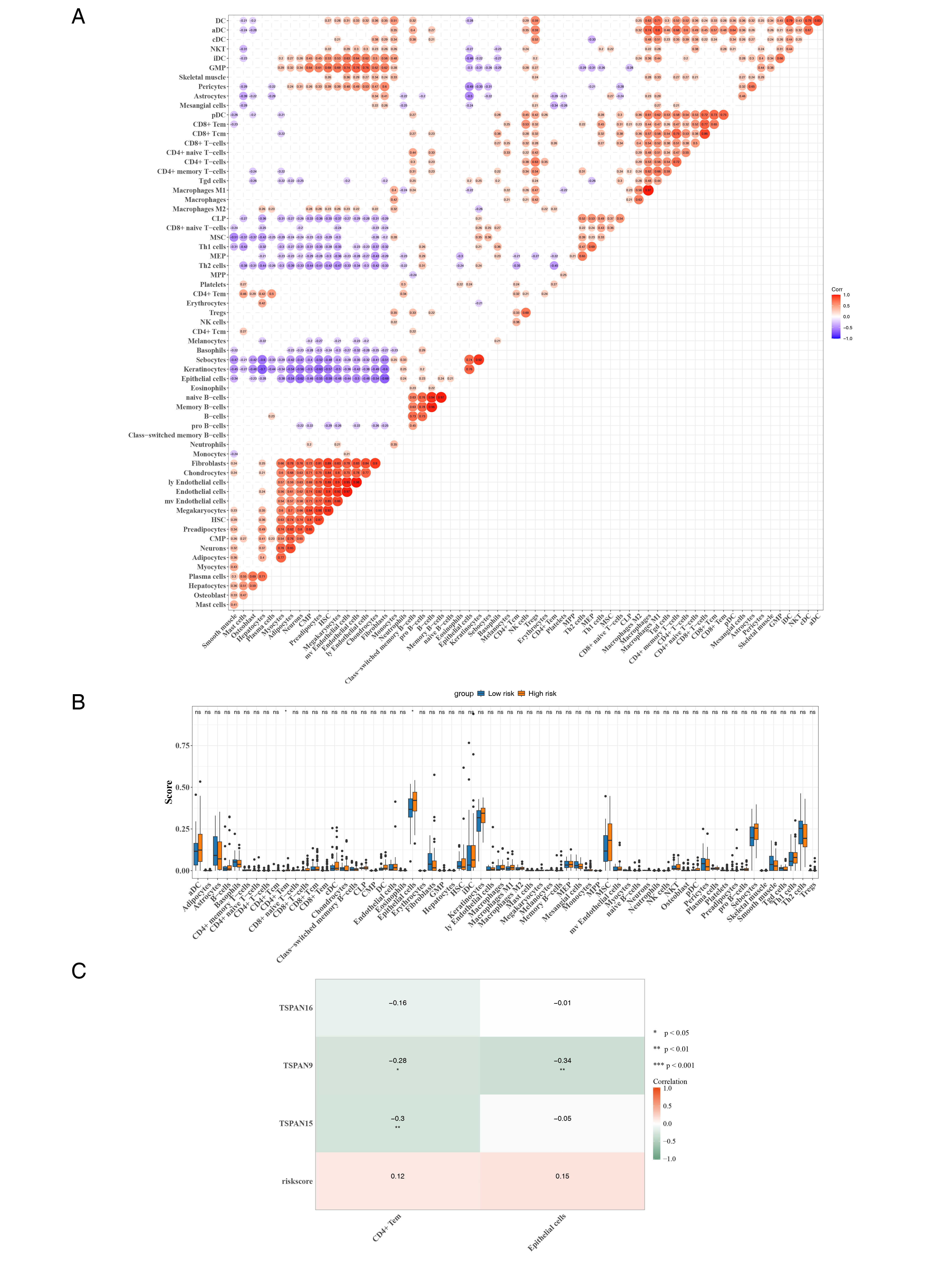

Supplement: Supplementary file 1 [file DataSheet1.zip › Supplementary Material/Supplementary Figure S4.tif]

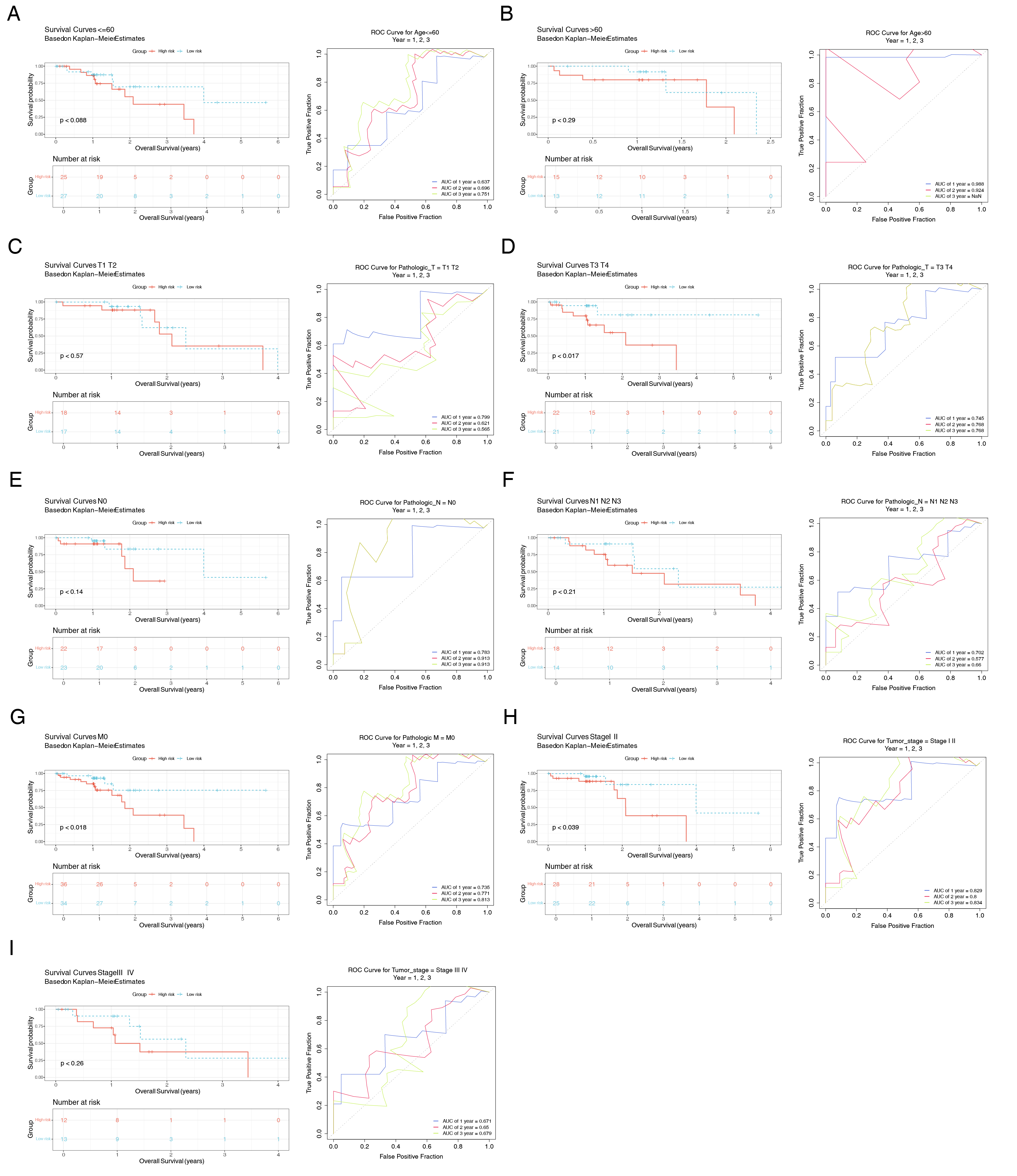

Supplement: Supplementary file 1 [file DataSheet1.zip › Supplementary Material/Supplementary Figure S2 .TIF]
